# An effective peptide vaccine strategy circumventing clonal MHC heterogeneity of murine myeloid leukaemia

**DOI:** 10.1038/s41416-020-0955-y

**Published:** 2020-06-29

**Authors:** A-Ri Shin, Sang-Eun Lee, Haeyoun Choi, Hyun-Jung Sohn, Hyun-Il Cho, Tai-Gyu Kim

**Affiliations:** 1 Department of Microbiology and Immunology, College of Medicine, The Catholic University of Korea, Seoul, 06591 South Korea; 2 Translational and Clinical Division, ViGenCell Inc., Seoul, 06591 South Korea; 3 Catholic Hematopoietic Stem Cell Bank, College of Medicine, The Catholic University of Korea, Seoul, 06591 South Korea

**Keywords:** Immunization, Cancer immunotherapy, Acute lymphocytic leukaemia

## Abstract

**Background:**

Therapeutic cancer vaccines are an attractive approach for treating malignant tumours, and successful tumour eradication depends primarily on controlling tumour immunosuppression status as well as heterogeneity of tumour cells driven by epigenetic alterations.

**Methods:**

Peptide-loaded dendritic cell (DC) prime and non-infectious peptide booster heterologous immunisations were assessed for the immunogenicity of polo-like kinase-1 (PLK1)-derived peptides. Heterologous vaccination regimen targeting multiple shared tumour antigens simultaneously with PD-L1 blockade was assessed against murine myeloid leukaemia.

**Results:**

A synthetic PLK1_122_ (DSDFVFVVL)-based heterologous vaccination generated large numbers of long-lasting antigen-specific CD8 T-cells eliciting therapeutic effects against various established tumours. The therapeutic efficacy of single antigen-targeting PLK1_122_-based vaccine with sufficient endurance of PD-L1 blockade toward C1498 leukaemia relied on the heterogeneous clonal levels of MHC-I and PD-L1 expression. A novel multi-peptide-based vaccination targeting PLK1 and survivin simultaneously along with PD1 blockade led to complete tumour eradication and long-term survival in mice with clonally heterologous C1498 myeloid leukaemia.

**Conclusions:**

Our findings suggest that PLK1 could be an attractive immunotherapeutic target antigen for cancer immunotherapy, and that similar strategies would be applicable for the optimisation of cancer vaccines for the treatment of numerous viral diseases and malignant tumours.

## Background

Substantial progress in cancer immunology has provided clear evidence that antigen-specific cytotoxic CD8 T-cells are key immune effectors that eliminate malignant tumour cells. However, in many instances, tumour-reactive CD8 T-cells are present in low frequency and/or lack the critical functionality to eradicate established tumours.^1,2^ Conceivably, most cancer immunotherapeutic strategies focus on unleashing tumour-reactive CD8 T-cell responses capable of recognising and destroying tumour cells. Nevertheless, the insufficient outcomes of current cancer immunotherapy can be largely attributed to the existence of immunosuppressive status such as elevated levels of T-cell inhibitory ligands, and to tumour heterogeneity that is driven by intrinsic factors such as genetic and/or epigenetic instability and alteration or loss of antigen expression eliciting reduced immunogenicity.^1,3–7^ Of these, blockade of T-cell inhibitory signals, especially PD-1/PD-L1 signal has achieved significant objective response rates correlated with intratumoural CD8 T-cell infiltration grade in the clinic.^8,9^ In contrast, tumour heterogeneity, mainly varied antigenicity remains a major challenge in the development of effective immunotherapies.

Several investigators including ours have attempted to utilise synthetic peptides representing CD8 T-cell epitopes as vaccines.^10–12^ Particularly, as targeting a single antigen may be inadequate to eradicate malignant tumour cells due to tumour escape driven by low and/or loss of target antigen, numerous reports have shown that targeting multiple epitopes from a single antigen or simultaneously targeting different antigens elicited a promising anti-tumour immunity in vivo.^13–15^ However, a significant long-term clinical benefit has been hardly achievable with these vaccines, indicating that their immunogenicity is suboptimal. Thus, there is a clear need for further optimising strategies to potentiate peptide-based vaccine, and for identifying and defining immunogenic novel T-cell epitopes to achieve the desired therapeutic benefit.

Since tumour cells are capable of altering targetable antigen expression to evade tumour immunity, we hypothesised that targeting survival-associated proteins such as shared antigens could be a way to overcome antigenic heterogeneity of tumours. Polo-like kinase-1 (PLK1), which is essential in cell-cycle regulation, is overexpressed in virtually all malignancies and its overexpression correlates with prognosis of cancer in patients.^16^ Considering this, PLK1 has been an attractive target for cancer drug development,^17,18^ and demonstrated to be a shared antigen useful for inducing T-cell responses.^19^ In this study, we evaluated the therapeutic benefits of a synthetic peptide PLK1_122_, corresponding to a CD8 T-cell epitope from PLK1 against myeloid leukaemia that exhibit a high level of tumour heterogeneity evolving upon disease progression and/or relapse.^20–22^ Moreover, we demonstrate that targeting multiple shared antigens,^23^ survivin (Sur) and PLK1 simultaneously in conjunction with prolonged blockade of checkpoint inhibitory signal could be used as an effective strategy to overcome antigenic heterogeneity, resulting in complete eradication of clonally heterogeneous C1498 myeloid leukaemia. Collectively, the present findings should facilitate the development of more effective immunotherapeutic strategies for tumours that may circumvent current limitations associated with cancer vaccines.

## Methods

### Mice and cell lines

C57BL/6 (female, 6–8-weeks-old) mice were purchased from Orient-Bio and maintained in our animal facilities under specific-pathogen-free conditions. Animal care and experiments were conducted according to our institutional animal-care and use committee guide-lines. Mice were anaesthetised with inhalation of 1.5–2% isoflurane and euthanised humanely by CO_2_ inhalation. Murine cell lines including C1498 leukaemia were obtained from the American Type Culture Collection and cultured as recommended by the provider. Luciferase-expressing C1498 transfectants (C1498-luc) were prepared using recombinant lentiviruses encoding firefly-luciferase-P2A-CD90.1 cDNA. After transduction, stable CD90.1-positive cells were isolated by flow cytometric sorting, and in some instances, C1498-luc clone was further isolated with cell cloning at limiting dilutions (referred as C1498^Homo^-luc).

### Peptides, antibodies and reagents

The peptide sequences used in this study are as listed in supplementary Table S1. All synthetic peptides were purchased at >80% purity from A&A Labs (San Diego, CA). All antibodies (Abs) for in vivo use including anti-PD-L1 (10F.9G2) and anti-PD-1 (29F.1A12) were from BioXCell (West Lebanon, USA). All fluorescence-conjugated Abs were from eBioscience (San Diego, CA).

### Immunisation

Dendritic cells (DCs) were prepared as described previously.^11^ For vaccination, mice were immunised intravenously with 2 × 10^6^ DCs pulsed with 10 µg/ml peptide(s) for 18 h. DCs were mixed with 30 µg poly-IC prior to injection, and 7-days later mice received an intravenous TriVax-boost immunisation consisting of 150 µg synthetic peptide(s), 50 µg poly-IC, and 100 µg anti-CD40 Abs. For PD-L1 blockade, anti-PD-L1 or anti-PD-1 Abs were administered intraperitoneally (200 µg/dose) after prime and boost immunisations twice on days +1 and +3, and for three-times on days +1, +3 and +5.

### Evaluation of immune responses

For measuring antigen-specific CD8 T-cell responses, peripheral blood samples or splenocytes were incubated with 1 μg/ml peptide and 1 μg/ml GolgiPlug (BD-Bioscience, San Diego, CA) at 37 °C. After 6 h, cells were stained for intracellular IFNγ following the directions provided by vendor (BD-Bioscience). For CD107a/b mobilisation assay, 2 μg/mL of fluorescence-conjugated anti-CD107a and CD107b Abs were added at the beginning of the stimulation period. To evaluate the in vitro T-cell tumour recognition, IFNγ-EliSpot assay were performed as described previously,^10^ using freshly isolated splenic CD8 T-cells (Miltenyi-Biotec, Germany). For peptide dose curve responses, serial peptide dilutions were incubated with RMA-S cells overnight and freshly purified CD8 T-cells were added for an additional 40 h before harvesting supernatants to measure IFNγ-production by ELISA (eBioscience).

### Anti-tumour effects

To assess the therapeutic effects, mice were inoculated subcutaneously with 2 × 10^6^ C1498-luc cells, and 7-days later when tumours measured >3–5 mm in diameter the first immunisation was administered. For orthotopic myeloid leukaemia settings, mice were inoculated intravenously with 2 × 10^6^ C1498-luc cells, and 7-days later, were given their first immunisation with peptide-loaded DCs. Seven-days later, the mice received a TriVax-booster immunisation. For in vivo depletion, each mouse received intraperitoneal administrations of 200 µg Abs against NK1.1, CD4, CD8, and CD25 on days −3 and −1 before the prime-boost immunisations. Depletions were confirmed with blood samples using flow cytometry (data not shown). The intravenous leukaemic tumour growth was assessed weekly by recording luciferase-bioluminescence from tumour tissue using Xenogen in vivo imaging system (Caliper Life-sciences, Hopkinton, MA), and presented the time course of in vivo bioluminescence of average radiance in individual mice.

### Single-cell preparation and real-time quantitative PCR

Livers and Lungs were dissected and chopped into 2–5 mm sizes and dissociated using the gentleMACS Dissociator following the directions provided by vendor (Miltenyi-Biotec). Intracellular co-staining of Foxp3 was conducted as per the instructions provided by vendor (eBioscience). The relative PLK1 expression levels in freshly isolate C1498 cells were measured using the TaqMan™ Gene Expression Master Mix (Thermo fisher, Massachusetts, USA) according to the manufacturer’s instruction. Sequences of primers and probes are as follows: PLK1 forward, 5′‐GCCTGAGGCCCGCTACTAC‐3′, and reverse, 5′‐TGATTGCGGTGCAGGTACTG‐3′; probe, 5′‐FAM‐TGCGACAGATAGTCCTG‐BHQ-1‐3′; β‐actin forward, 5′‐CGATGCCCTGAGGCTCTTT‐3′ and reverse, 5′‐TGGATGCCACAGGATTCCA‐3′; probe, 5′‐FAM‐CCAGCCTTCCTTCTT‐BHQ-1‐3′. The levels of PLK1 gene expression divided by those of β‐actin gene expression were defined as relative PLK1 expression.

### Statistical analyses

Results are representative of data obtained from at least two independent experiments. Statistical significance to assess the numbers of antigen-specific CD8 T-cells was determined by unpaired Student’s *t-*tests, and survival analysis was established by Kaplan–Meier curves using log-rank tests. All analyses were performed using Prism 5.01 software (GraphPad).

## Results

### Immune responses to PLK1-derived peptide in C57BL/6 mice

**Fig. 1:**
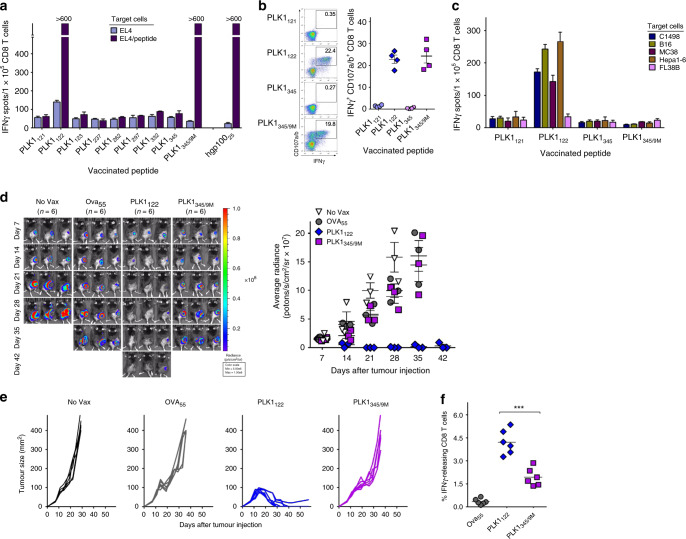
Immunisation with PLK1 peptides induces CD8 T-cell responses resulting in potent anti-tumour immunity. B6 mice (2 per group) were immunised intravenously with 2 × 10^6^ DCs loaded with the indicated synthetic peptide (prime); 7-days later, the mice received a booster immunisation with TriVax composed of 100 μg of peptide, 50 μg of poly-IC, and 100 μg of anti-CD40 Abs. **a** Eight-days after the boost, CD8 T-cells were purified from pooled splenocytes, and antigen-induced IFNγ secretion toward each corresponding peptide was evaluated using EliSpot assay. Peptide hgp100_25_ (KVPRNQDWL) were incorporated for comparison. Wherever the number of spots are too numerous to count, they are shown as >600. Results represent the average number of spots from triplicate wells with SD (*bars*) of the means. **b** Frequency of peptide-specific CD8 T-cells in spleen from PLK1_121_-, PLK1_122_-, PLK1_345_-, or PLK1_345/9M_-immunised individual mouse was evaluated by cell surface mobilisation of CD107a/b and intracellular IFNγ staining after coculturing with corresponding peptide. A representative dot plot analysis for one mouse of each group is presented (*left panel*). Numbers in each rectangular gate represent the % IFNγ and cell surface CD107a/b double-positive cells of all CD8 T-cells. The results are the sum of two independent experiments with two per group. *Points*, values for each individual mouse; *bars*, SD. **c** Freshly isolated CD8 T-cells from pooled splenocytes in **b** were evaluated for antigen-induced IFNγ secretions by EliSpot against various tumour cell lines C1498 (leukaemia), B16 (melanoma), MC38 (colon carcinoma), and Hepa1-6 (hepatoma). Results represent the average number of spots from triplicate wells with SD (*bars*) of the means. Normal liver cell line FL83B was included as a negative control. **d**–**f** Therapeutic efficacy of PLK1_122_ or PLK1_345/9M_-based vaccination as in **a** against subcutaneously established C1498 leukaemia. B6 mice (6 per group) were inoculated subcutaneously with 2 × 10^6^ C1498-luc cells on day 0 and received PLK1_122_ or PLK1_345/9M_-loaded DC prime_TriVax boost immunisation on day 7 and 14, followed by weekly bioluminescence imaging. Non-vaccinated mice (No Vax) and Ova_55_-loaded DCs prime_TriVax booster-vaccinated mice (Ova_55_) were included as controls. **d** Tumour growth was monitored by time course of in vivo bioluminescence imaging in individual mice (*left panel*), and average radiance per mouse is shown (*right panel*). Images were adjusted to the same pseudo colour scale to show relative bioluminescence changes over time. *Points*, average values of photons in mouse; *bars*, SD. **e** Each line corresponds to the tumour size of each individual mouse. Tumour sizes were measured by the two opposing diameters and are presented as tumour areas in mm^2^. **f** Frequency of PLK1_122_-specific CD8 T-cells was evaluated by intracellular IFNγ staining on day 32 using blood samples from mice in **d**. *Points*, value for each individual mouse; *horizontal line*, average of the group. *P* values were calculated using unpaired Student’s *t* test (***, *P* < 0.001). These experiments were repeated twice with similar results.

First, we sought to identify at least one PLK1-derived CD8 T-cell epitope that could be naturally processed and presented on tumour cells, and thus be used for a peptide-based cancer therapy. Using three web-based MHC-peptide prediction algorithms, PLK1 was analysed for the presence of H-2^b^-binding motifs. Eight peptide sequences and a mutated peptide PLK1_345/9M_, which has a substitution at position 9M for D to improve MHC-I binding affinity, were selected (Table S1). To assess the immunogenicity of the peptides, mice were individually vaccinated using a peptide-loaded DCs prime and TriVax boost immunisation regimen, which was highly efficient in stimulating and expanding antigen-specific CD8 T-cells in mice.^11^ As shown in Fig. 1a, peptides PLK1_122_ and PLK1_345/9M_ were found to induce antigen-specific CD8 T-cell responses, which was similar to that observed using a well-known hgp100_25_ CD8 T-cell epitope. Furthermore, we observed the capacity of PLK1_122_ and PLK1_345/9M_ to bind to empty H-2K^b^ and H-2D^b^, respectively, and PLK1_122_ was more effective in binding to H-2K^b^ in comparison to the positive control, H-2K^b^-binder Trp2_180_ (Fig. S1A). PLK1_345/9M_ had lower MHC-I dissociation rates in comparison to H-2D^b^-binder hgp100_25_ peptide. PLK1_122_ had fast dissociation rates at early time points but higher amounts of PLK1_122_ remained bound to H-2K^b^ in comparison to the H-2K^b^-bound Trp2_180_ (Fig. S1B). Then, peptides PLK1_121_, PLK1_122_, PLK1_345_, and PLK1_345/9M_ were evaluated for their capacity to induce CD8 T-cell responses capable of recognising PLK1-expressing tumour cells. PLK1_122_ and PLK1_345/9M_ generated higher number of antigen-specific IFNγ-producing CD8 T-cells with lytic functionality (CD107a/b mobilisation) in comparison to that generated by PLK1_121_ and PLK1_345_ (Fig. 1b). However, as shown in Fig. 1c, only the freshly isolated CD8 T-cells from PLK1_122_-vaccinated mice displayed high recognition activity against PLK1-expressing tumour cells without reacting to PLK1-negative cells, which were confirmed with a western blot (Fig. S1C).

### Therapeutic anti-tumour effects of PLK1-derived peptide vaccination

Next, we evaluated whether PLK1-based peptide vaccination would offer a therapeutic benefit in vivo against established syngeneic tumours. Mice were first challenged subcutaneously with C1498-luc, and 7-days later, when tumours reached a size of 3–5 mm diameter the mice received PLK1-derived peptide-loaded DC prime_TriVax booster immunisation with a 7-day interval. Although PLK1_345/9M_ and Ova_55_DC_TriVax had moderate therapeutic effects when compared to unvaccinated mice, mice that received PLK1_122_DC_TriVax vaccination had substantially reduced tumour progression as compared to those with PKL1_345/9M_ and Ova_55_ peptides, marked by significantly lower bioluminescent signals (by day42), which correlated with the observed tumour growth (5 out of 6 mice completely rejected; Fig. 1d, e) and the levels of antigen-specific CD8 T-cells observed in blood (Fig. 1f). To validate the reproducibility and durability of anti-tumour immunity, mice that had rejected tumours were re-challenged (on day49) with the same C1498-luc cells without any further treatment and were monitored for tumour growth. None of these mice developed tumours, in contrast to a naïve control group challenged with the same tumour, implying that PLK1_122_DC_TriVax immunisation could induce long-term antigen-specific memory CD8 T-cell responses (Fig. S2). Likewise, we verified that PLK1_122_DC_TriVax vaccination could extend to another 7-day-established subcutaneous Hepa1-6 hepatoma, where tumour was completely eradicated (Fig. S3A, B). Notably, when the mice that rejected Hepa1-6 tumours were re-challenged on day48 with C1498-luc cells, the mice could reject the secondary tumour challenge (Fig. S3C, D). So far, these results imply that PLK1 protein can be used as a shared tumour antigen and PLK1_122_ is the most effective peptide in generating CD8 T-cells capable of recognising numerous PLK1-expressing tumour cells, resulting in potent therapeutic anti-tumour effects.

**Fig. 2:**
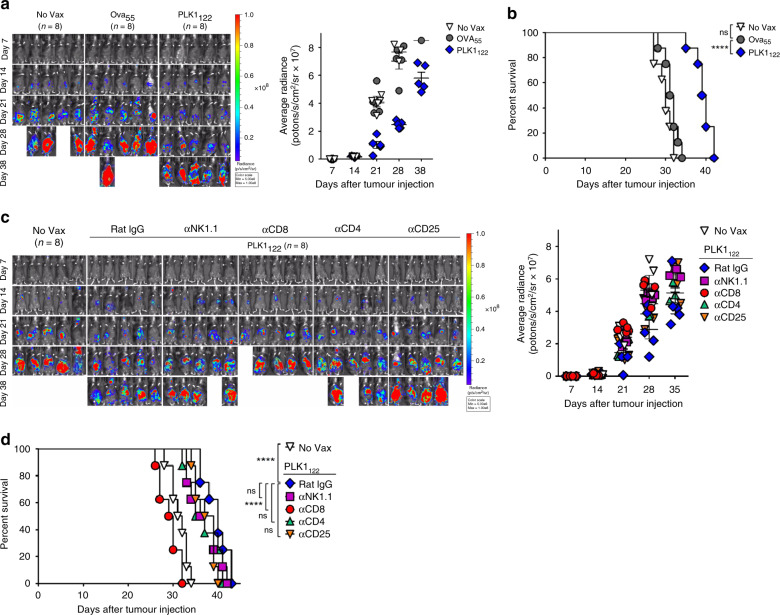
Therapeutic anti-tumour effects of PLK1_122_DC_TriVax vaccination against murine C1498 leukaemia. B6 mice (8 per group) received intravenously 2 × 10^6^ C1498-luc cells on day 0 and received PLK1_122_-loaded DC prime_TriVax boost immunisation on day 7 and 14, followed by weekly bioluminescence imaging. **a** Therapeutic efficacy of PLK1_122_-based vaccination against intravenously established C1498 leukaemia. Non-vaccinated mice (No Vax) and Ova_55_-loaded DCs prime_TriVax booster-vaccinated mice (Ova_55_) were included as controls. **c** Effector mechanism involved in anti-tumour effects. C1498-induced leukaemic mice were administered with indicated antibodies on days −1 and −3 before immunisations. No antibody-treated (No Tx) mice were included as controls. **a**, **c** Tumour growth was monitored by time course of in vivo bioluminescence imaging in individual mice (*left panel*), and average radiance per mouse is shown (*right panel*). Images were adjusted to the same pseudo colour scale to show relative bioluminescence changes over time. *Points*, average values of photons in mouse; *bars*, SD. **b**, **d** Kaplan–Mayer survival curves for all groups of mice in **a**, **c**, respectively. *P* values were determined by log-rank tests (ns, not significant; ****, *P* < 0.0001). These experiments were repeated twice with similar results.

Next, we demonstrated the therapeutic efficacy of PLK1_122_DC_TriVax vaccination regime in orthotopic C1498 myeloid leukaemia, which offer more clinically relevant tissue site-specific tumour setting. Mice were intravenously engrafted with C1498-luc cells, and 7-days later, the PLK1_122_DC_TriVax vaccinations were initiated. As shown in Fig. 2a, Ova_55_DC_TriVax had moderate therapeutic effects as compared to unvaccinated mice, whereas mice receiving PLK1_122_DC_TriVax had substantially reduced tumour progression in comparison to those with Ova_55_ peptides, resulted in significantly increased median survival of the mice by more than at least 1-week (Fig. 2b). Subsequently, we assessed the roles of CD8 T-cells, CD4 T-cells, regulatory T-cells and NK-cells in controlling tumour progression by in vivo depletion. Elimination of CD8 T-cells completely abolished the therapeutic benefit of PLK1_122_DC_TriVax vaccination, indicating that CD8 T-cells are crucial for the controlling of established C1498 myeloid leukaemia (Fig. 2c, d). Depletion of other cells had moderate, but not significant effects on the therapeutic benefit, indicating that these cells may somehow contribute to inhibit tumour growth.

### PD-L1 blockade augments the therapeutic efficacy of PLK1_122_-based vaccination

**Fig. 3:**
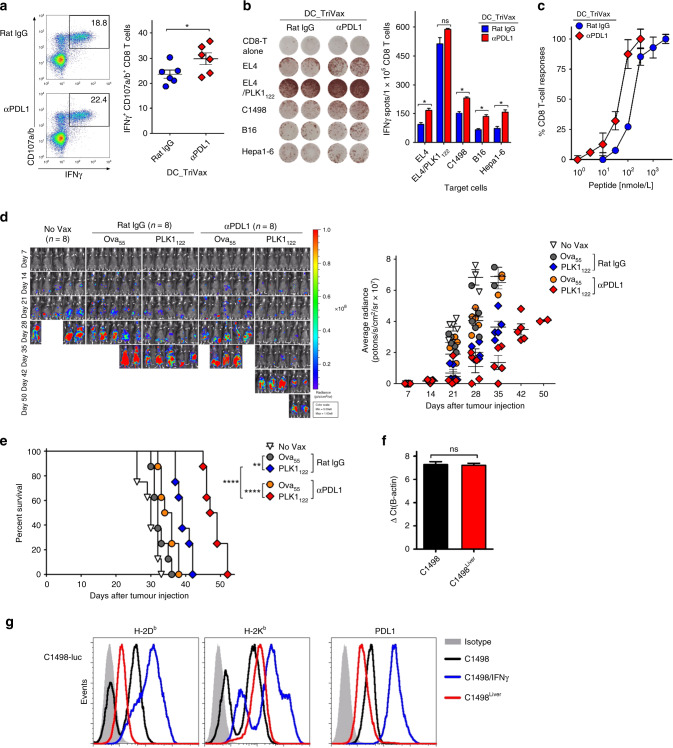
PD-L1 blockade enhance the therapeutic efficacy of PLK1_122_DC_TriVax vaccination against murine C1498 leukaemia. **a**–**c** Effects of PD-1 blockade on the capacity of PLK1_122_-specific CD8 T-cells to recognise tumour cells. B6 mice (3 per group) were immunised intravenously with PLK1_122_DCs_TriVax on day 7 and 14 with and without anti-PD-L1 Abs. Anti-PD-L1 and normal rat IgG were administered twice intraperitoneally (200 μg/dose) on days +1 and +3 after each immunisation. **a** Eight-days after the boost, frequency of PLK1_122_-specific CD8 T-cells in spleen was evaluated by cell surface mobilisation of CD107a/b and intracellular IFNγ staining as in Fig. 1b. A representative dot plot analysis for one mouse from each group is represented (*left panel*). Numbers in each rectangular gate represent the % IFNγ and cell surface CD107a/b double-positive cells of all CD8 T-cells. The results are the sum of two independent experiments with triplicate per group. *Points*, values for each mouse; *bars*, SD. **b** Freshly isolated CD8 T-cells from pooled splenocytes in **a** were evaluated for antigen-induced IFNγ secretions by EliSpot against indicated target cells. Photos represent examples of wells obtained using 1 × 10^5^ CD8 T-cells and 1 × 10^5^ tumour cells per well (*left panel*). Results represent the average number of spots from triplicate wells with SD (*bars*) of the means. *P* values were calculated using unpaired Student’s *t* tests (ns, not significant; *, *P* < 0.05; **, *P* < 0.01). **c** Antigen-dose responses of purified CD8 T-cells isolated from mice immunised with PLK1_122_ peptide under PD-L1 blockade. Results represent the percent T-cell response compared to the maximal response (100%) for each T-cell line with SD (*bars*) from triplicate cultures. **d**, **e** Therapeutic efficacy of PLK1_122_-based vaccination with PD-L1 blockade against C1498 leukaemia. B6 mice (8 per group) received intravenously 2 × 10^6^ C1498-luc cells and received PLK1_122_DC_TriVax immunisation with and without anti-PD-L1 mAb as in **a**. Non-vaccinated mice (No Vax) and Ova_55_-DC_TriVax-vaccinated mice (Ova_55_) were included as controls. **d** Tumour growth was monitored by time course of in vivo bioluminescence imaging in individual mice (*left panel*), and average radiance per mouse is shown (*right panel*). Images were adjusted to the same pseudo colour scale to show relative bioluminescence changes over time. *Points*, average values of photons in mouse; *bars*, SD. **e** Kaplan–Mayer survival curves for all groups of mice in **d**. *P* values were determined by log-rank tests (**, *P* < 0.01; ****, *P* < 0.0001). **f**, **g** Comparison of the expression levels of PLK1 protein (**f**), surface of MHC-I (H-2D^b^ and H-2K^b^), and surface of PD-L1(**g**) in maternal C1498 and in vivo-isolated C1498-luc (C1498^Liver^). C1498^Liver^ cells were isolated from liver tissue of mice that received PLK1_122_DC_TriVax with PD-L1 blockade after 52-days of tumour infusion. **f** Relative PLK1 mRNA expression levels in C1498 leukaemia tumours were determined by real-time quantitative-PCR. **g** The maternal C1498-luc cells were incubated with and without 100 IU/ml IFNγ for 40 h for comparison, and freshly isolated C1498^Liver^ cells were immediately (without additional treatment) analysed by flow cytometry after staining with specific antibodies. These experiments were repeated twice with similar results.

Subsequently, we evaluated the effect of the addition of anti-PD-L1 Abs (on days +1 and +3 after each immunisation) to the PLK1_122_DC_TriVax vaccination regimen. As shown in Fig. 3a, PLK1_122_DC_TriVax administered along with anti-PD-L1 Abs generated evidently higher number of antigen-specific IFNγ-producing CD8 T-cells as compared to those with isotype control rat-IgG, which correlated with the functional activity capable of recognising not only against PLK1_122_-pulsed targets but also toward numerous tumour cells (Fig. 3b). A peptide titration curve comparison between these CD8 T-cells revealed that the PLK1_122_-specific CD8 T-cells induced in presence of PD-L1 blockade exhibited an approximately 10-fold higher avidity in comparison to CD8 T-cells generated in absence of PD-L1 blockade (Fig. 3c). Next, we assessed whether in vivo PD-L1 blockade could potentiate the therapeutic efficacy of PLK1_122_DC_TriVax vaccination in a more clinically relevant myeloid leukaemia setting. As shown in Fig. 3d, e, the addition of PD-L1 blockade in PLK1_122_DC_TriVax led to dramatically reduced tumour progression and significantly increased median survival of the mice by more than 1-week as compared to those with PLK1_122_DC_TriVax alone, but complete tumour regressions were not obtained. Ova_55_DC_TriVax had moderate effects in comparison to unvaccinated mice and the administration of isotype rat-IgG had no effects on tumour progression.

To further study any issues that impeded the efficacy of the combination of PD-L1 blockade with PLK1_122_DC_TriVax in myeloid leukaemia setting, we initially assessed for the presence of antigen-specific CD8 T-cells after euthanatising three mice in each group after 23-days of tumour infusion. As shown in Fig. S4A, B, mice that received the PLK1_122_DC_TriVax with PD-L1 blockade had comparably higher numbers of antigen-specific CD8 T-cells capable of effectively recognising C1498 tumour cells, whereas mice that received the Ova_55_DC_TriVax did not have any detectable PLK1_122_-specific CD8 T-cell responses. In this circumstance, we further investigated the presence of C1498 tumour cells, CD8 T-cell expansion, and the frequency of myeloid-derived suppressor cells (MDSC) in different tissues. The ratios of CD8 T-cells to regulatory T-cell and the frequency of CD90.1^+^C1498 cells were inversely correlated in several tissues (Fig. S4C, D). The combined treatment of PD-L1 blockade with PLK1_122_DC_TriVax led to decreased levels of Ly6G^low^Ly6C^high^ monocytic MDSC subsets, but not Ly6G^high^Ly6C^low^ granulocytic polymorphonuclear MDSC, which were presented as percentage of CD11b^+^CD115^high^ myeloid cells in the livers and lungs (Fig S4E–G). Likewise, histochemical analysis also evidently displayed cancerous blast suppression in mice that received PLK1_122_ vaccinations in all tissues, except lungs (Fig. S4H).

### Effects of prolonged period of PD-L1 blockade on PLK1_122_-based vaccination

In our further studies involving PD-L1 blockade, we evaluated the duration period of the combinatorial Abs delivery and the alternative efficacy of anti-PD-1 Abs since its blocking effect could also be achieved by hindering PD-1 receptors that are expressed on T-cells. We compared the efficacy of three doses of anti–PD-1 and anti–PD-L1 Abs (on days +1, +3, and +5 after each immunisation) in combination with PLK1_122_DC_TriVax vaccination in same leukaemia settings. Surprisingly. the prolonged period of PD-L1 blockade dramatically enhanced the therapeutic efficacy of PLK1_122_DC_TriVax, in which complete tumour regressions were attained (two out of eight mice; Fig. S5A, B). In contrast, although moderate therapeutic effects were observed with anti-PD-1 Abs as compared to those with Ova_55_ peptides, no significant improvement of tumour regression was observed in comparison to that observed with anti–PD-L1 Abs. Particularly, at day 72, freshly isolated CD8 T-cells from survivor mice exhibited effective recognition not only to PLK1_122_-pulsed targets but also towards C1498 tumour cells (Fig. S5C), suggesting that the sufficient endurance of PD-L1 blockade could potentiate the therapeutic effectiveness of PLK1_122_DC_TriVax to C1498 myeloid leukaemia.

Moreover, since tumour cells with decreased antigenicity could evade immune-mediated elimination and promote tumour outgrowth, resulting in tumour clonal heterogeneity,^24^ we examined the expression levels of PLK1 protein, surface MHC-I, and surface PD-L1 on freshly isolated CD90.1^+^C1498 cells from liver tissue (referred to as C1498^Liver^) of mice that exhibited progressive tumour growth after receiving PLK1_122_DC_TriVax with PD-L1 blockade post 52-days of tumour infusion. As shown in Fig. 3f, there was no difference in the expression of PLK1 mRNA between maternal C1498-luc that was used in previous experiments and in vivo-isolated C1498^Liver^. However, when investigating the surface expression of MHC-I (H-2D^b^ and H-2K^b^) and PD-L1, the maternal C1498-luc revealed heterogeneous levels of MHC-I and relatively high level of homogenous PD-L1 expression, and these molecules were upregulated in response to inflammatory stimuli IFNγ that could be considered as inflammatory conditions of tumour sites in vivo (Fig. 3g). More interestingly however, freshly isolated C1498^Liver^ cells exhibited decreased homogenous levels of MHC-I and PD-L1 in comparison to IFNγ-treated maternal C1498-luc cells.

### Multi-antigenic peptide-based vaccination with sustained PD-L1 blockade circumvents antigenic heterogeneity of myeloid leukaemia

**Fig. 4:**
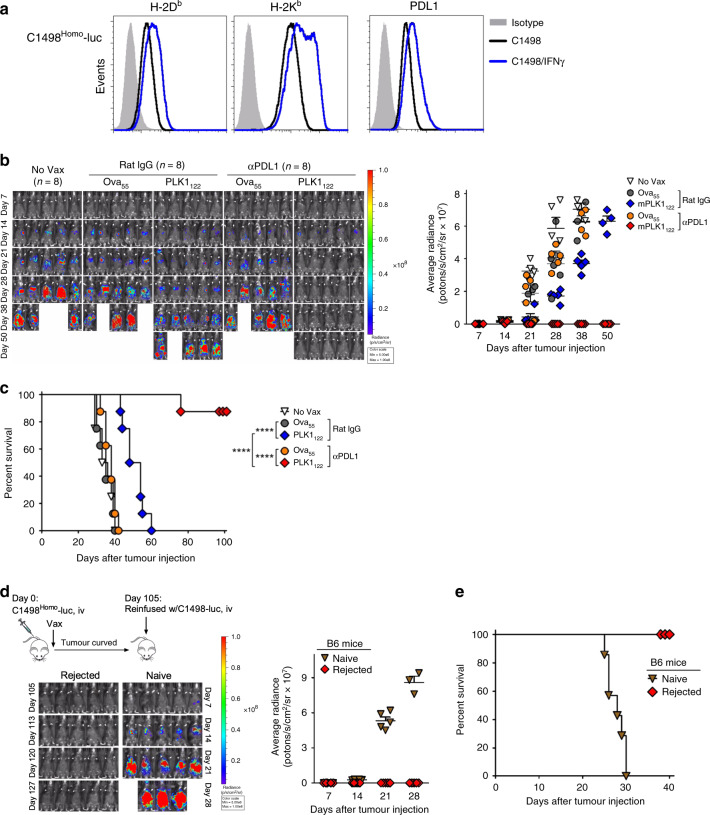
PLK1_122_DC_TriVax immunisation with PD-L1 blockade induces long-lasting CD8 T-cell responses capable of eradicating homogenous C1498^Homo^-luc leukaemia. **a** Expression levels of MHC-I (H-2D^b^ and H-2K^b^) and PD-L1 on C1498^Homo^-luc cells, which were sub-clonally isolated from maternal C1498-luc cells. The cells were incubated with and without 100 IU/ml IFNγ for 40 h and analysed by flow cytometry after staining with specific antibodies. **b**, **c** B6 mice (8 per group) were inoculated intravenously with 2 × 10^6^ C1498^Homo^-luc cells at day 0, and the mice were treated in the same manner as in Fig. 3d, followed by weekly bioluminescence imaging. Non-vaccinated mice (No Vax) and Ova_55_DC_TriVax-vaccinated mice (Ova_55_) were included as controls. Anti-PD-L1 and normal rat IgG were administered twice intraperitoneally (200 μg/dose) on days +1 and +3 after each immunisation. **d**, **e** 105-days later, tumour-free (rejected) survivor mice from **b** received intravenous tumour rechallenge with 2 × 10^6^ maternal clonally heterogeneous C1498-luc cells. Naive, unvaccinated mice inoculated with the same number of C1498-luc cells were incorporated for comparison. **b**, **d**. Tumour growth was monitored by time course of in vivo bioluminescence imaging in individual mice (*left panel*), and average radiance per mouse is shown (*right panel*). Images were adjusted to the same pseudo colour scale to show changes in relative bioluminescence over time. *Points*, average values of photons in mouse; *bars*, SD. **c**, **e** Kaplan–Mayer survival curves for all groups of mice in **b**, **d**, respectively. *P* values were determined by log-rank tests (****, *P* < 0.0001). These experiments were repeated twice with similar results.

The above observations suggest that the tumour recognition of T-cells and the efficacy of anti–PD-L1 Abs depend on the expression levels of MHC-I and PD-L1 molecules, and down-regulation of MHC-I and PD-L1 expression could be result of in vivo immune-escaping caused by heterogeneous antigenic expression of C1498 leukaemic cells. Thus we hypothesised that C1498-luc that express homogenous level of H-2K^b^ could be more susceptible to PLK1_122_DC_TriVax with PD-L1 blockade therapeutic regime, and that superior therapeutic effects against clonally heterogeneous C1498 leukaemia could be achieved with multi-antigen-specific CD8 T-cells targeting multi-epitopes bound to both MHC-I allele, H-2D^b^ and H-2K^b^ to attenuate in vivo-escaping of tumour cell clones. To test the first hypothesis, C1498^Homo^ were isolated from heterogeneous C1498-luc, which expressed homogenous levels of MHC-I and PD-L1 molecules (Fig. 4a). Then, we assessed the therapeutic effectiveness of PLK1_122_DC_TriVax against 7-day-engrafted C1498^Homo^ leukaemia with or without anti-PD-L1 Abs. As shown in Fig. 4b, c, PLK1_122_DC_TriVax alone had moderate effects in comparison to those with Ova_55_ peptides, whereas complete tumour regression was accomplished in the majority of the mice (seven out of eight mice) when administered in combination with anti-PD-L1 Abs. To verify the long-term anti-tumour immunity, the survivor mice were re-challenged (on day 105) with maternal heterogeneous C1498-luc cells without any further treatment. None of these mice developed tumours, in contrast to a naïve control group challenged with the same tumour (Fig. 4d, e), In consistent with our hypothesis, these results imply that the limiting of the therapeutic efficacy of PLK1_122_DC_TriVax is mediated mostly through the heterogeneous antigenic presentation on MHC-I caused by heterogeneity of tumour cell clones.

**Fig. 5:**
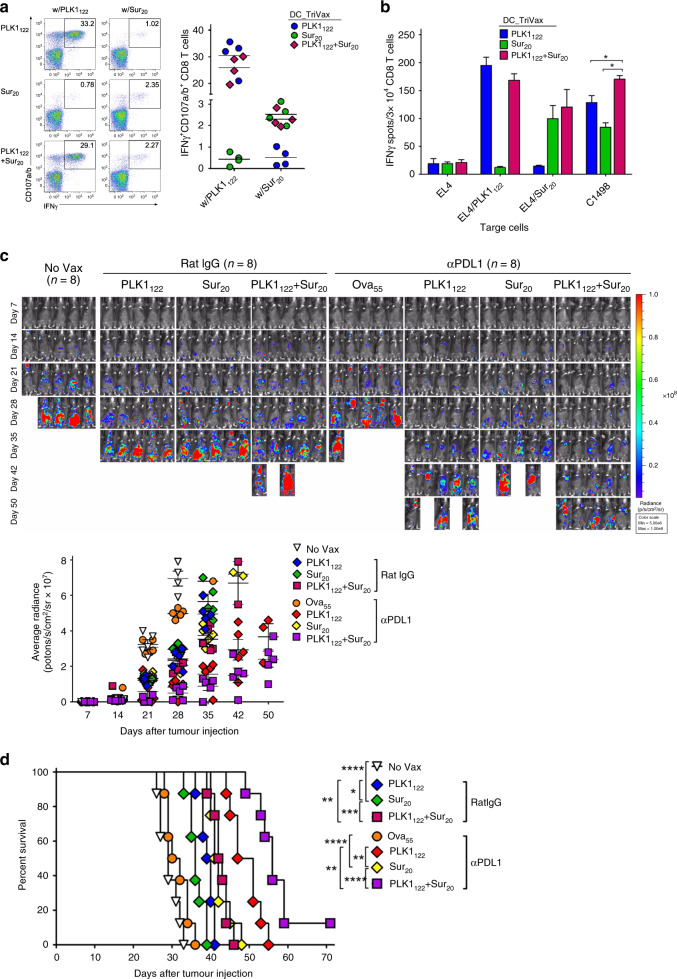
Multi-antigenic peptide vaccine comprising PLK1_122_ and Sur_20_ induces concurrent CD8 T-cell responses capable of enhancing the therapeutic anti-tumour efficacy with PD-L1 blockade. **a**, **b** B6 mice (2 per group) were vaccinated intravenously with DCs loaded with PLK1_122_ and Sur_20_ (ATFKNWPFL) either individually or in pairs as indicated (*prime*); 7-days later, the mice received booster immunisation with the identical peptide-TriVax. **a** Eight-days after the boost, the frequency of PLK1_122_-specific CD8 T-cells in spleen was evaluated by cell surface mobilisation of CD107a/b and intracellular IFNγ staining as in Fig. 1b. A representative dot plot analysis for one mouse of each group is presented (*left panel*). Numbers in each rectangular gate represent the % IFNγ and cell surface CD107a/b double-positive cells of all CD8 T-cells. The results are the sum of two independent experiments. *Points*, values for each mouse; *bars*, SD. **b** Freshly isolated CD8 T-cells from pooled splenocytes in **a** were evaluated for antigen-induced IFNγ secretions by EliSpot against indicated target cells. Results represent the average number of spots from triplicate wells with SD (*bars*) of the means. *P* values were calculated using unpaired Student’s *t* test (*, *P* < 0.05). **c** PD-L1 blockade enhance the therapeutic efficacy of multi-antigenic peptide-based vaccination against C1498-luc leukaemia. B6 mice (8 per group) received intravenously 2 × 10^6^ C1498-luc cells and received DC_TriVax immunisation in the same manner as in **a** with and without administration of anti-PD-L1 Abs, followed by weekly bioluminescence imaging. Non-vaccinated mice (No Vax) and Ova_55_DC_TriVax-vaccinated mice (Ova_55_) were included as controls. Anti-PD-L1 and normal rat IgG were administered twice intraperitoneally (200 μg/dose) on days +1 and +3 after each immunisation. Tumour growth was monitored by time course of in vivo bioluminescence imaging in individual mice (*upper panel*), and average radiance per mouse is shown (*lower panel*). Images were adjusted to the same pseudo colour scale to show relative bioluminescence changes over time. *Points*, average values of photons in mouse; *bars*, SD. **d** Kaplan–Mayer survival curves for all groups of mice in **c**. *P* values were determined by log-rank tests (*, *P* < 0.05; **, *P* < 0.01; ***, *P* < 0.001; ****, *P* < 0.0001). These experiments were repeated twice with similar results.

Next, to validate the second possibility, Sur_20_ (ATFKNWPFL) peptide, known as naturally processed H-2D^b^ epitope^25^ was incorporated with PLK1_122_ because we sought to demonstrate our hypothesis by targeting clinically relevant shared antigens. As shown in Fig. 5a, DC_TriVax using Sur_20_ generated quite low numbers of antigen-specific lytic-functional CD8 T-cells in comparison to those generated by PLK1_122_, whereas multi-peptides PLK1_122_/Sur_20_DC_TriVax triggered simultaneous multivalent CD8 T-cells at levels comparable to that triggered by the administration of individual peptides. Correspondingly, freshly isolated splenic CD8 T-cells from PLK1_122_/Sur_20_-immunised mice recognised C1498 tumour cells at significantly better efficiency in comparison to their recognition by CD8 T-cells from individual peptide-immunised mice (Fig. 5b). Subsequently, we evaluated the therapeutic efficacy of PLK1_122_/Sur_20_DC_TriVax against clonally heterogeneous C1498-luc-engrafted leukaemia in comparison with the individual PLK1_122_- or Sur_20_DC_TriVax immunisations. As shown in Fig. 5c, d, administration of Sur_20_DC_TriVax had considerable effects resulting in extended survival rates of mice in comparison to PLK1_122_DC_TriVax with PD-L1 blockade. Notably, multi-peptide PLK1_122_/Sur_20_DC_TriVax immunisation combining PD-L1 blockade exhibited significantly superior therapeutic anti-tumour effects, where complete tumour eradication was attained (one out of eight mice). The considerably improved effectiveness of PLK1_122_/Sur_20_DC_TriVax was also obtained even in the absence of PD-L1 blockade.

**Fig. 6:**
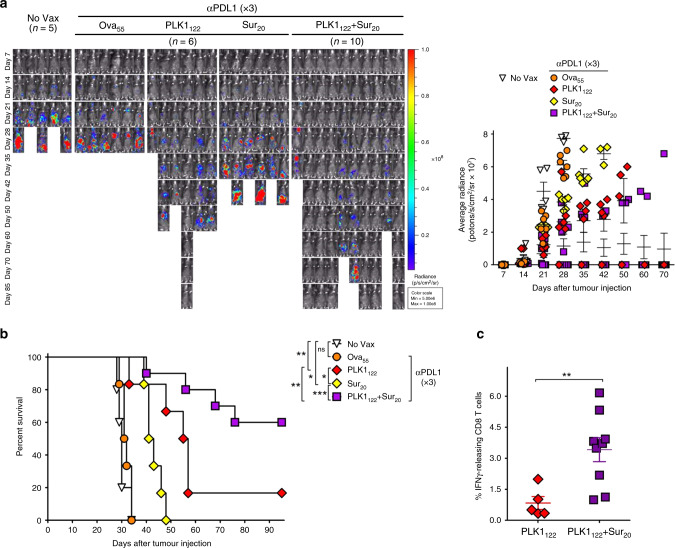
DCs priming followed by TriVax boost with multi-peptides with sustained PD-L1 blockade elicits augmented therapeutic effectiveness overcoming clonal heterogeneity of C1498 leukaemia. B6 mice (6–10 per group) were intravenously 2 × 10^6^ C1498-luc cells and received DC_TriVax immunisation using either individual peptide or mixture of PLK1_122_ and Sur_20_ (as indicated) with prolonged period of PD-L1 blockade, followed by weekly bioluminescence imaging. Non-vaccinated mice (No Vax) and Ova_55_DC_TriVax-vaccinated mice (Ova_55_) were included as controls. Anti-PD-L1 was administered three-times (×3) on days +1, +3 and +5 for long-term treatment after each immunisation. **a** Tumour growth was monitored by time course of in vivo bioluminescence imaging in individual mice (*left panel*), and average radiance per mouse is shown (*right panel*). Images were adjusted to the same pseudo colour scale to show relative bioluminescence changes over time. *Points*, average values of photons in mouse; *bars*, SD. **b** Kaplan–Mayer survival curves for all groups of mice in **a***. P* values were determined by log-rank tests (ns,  not significant; *, *P* < 0.05; **, *P* < 0.01; ***, *P* < 0.001). **c** Frequency of PLK1_122_-specific CD8 T-cells was evaluated by intracellular IFNγ staining on day 45 using blood samples from mice in **a**. *Points*, value for each individual mouse; *horizontal line*, average of the group. *P* values were calculated using unpaired Student’s *t* test (***, *P* < 0.01). These experiments were repeated twice with similar results.

Lastly, we assessed the therapeutic efficacy of sustained PD-L1 blockade combined with PLK1_122_/Sur_20_DC_TriVax in same leukaemia settings. The results presented in Fig. 6a, b conclude that PLK1_122_/Sur_20_DC_TriVax immunisation was effective in completely eradicating clonally heterogeneous C1498-luc leukaemia in the majority of the mice when administered in combination with prolonged period of PD-L1 blockade (6 out of 10 mice survived). Remarkably, PLK1_122_/Sur_20_DC_TriVax with PD-L1 blockade revealed higher numbers of PLK1_122_-specific CD8 T-cells as compared to using PLK1_122_DC_TriVax (Fig. 6c). Moreover, since the disease stage may affect the T-cell immunity, PLK1_122_/Sur_20_DC_TriVax vaccination regimen was applied to treat 14-day-engrafted (more advanced) C1498 myeloid leukaemia. Under these circumstances, the sustained PD1 blockade with PLK1_122_/Sur_20_DC_TriVax reduced the median tumour growth rate significantly, but no tumour rejections were observed (Fig. S6). These overall results suggest that the induction of multiple antigen-targeting CD8 T-cells and sufficient endurance of PD-L1 blockade amplified intensely the therapeutic effectiveness of DC_TriVax regimen towards clonally heterogeneous C1498 myeloid leukaemia.

## Discussion

Over the past decades, the identification of tumour antigens for T-cells and their corresponding T-cell epitopes have expedited development of peptide-based vaccines against malignant tumours. In this study, our original intention was to evaluate the therapeutic efficacy of the combined immunotherapeutic strategy of peptide-based DCs prime_TriVax booster regimen with PD-L1 blockade in orthotopically established myeloid leukaemia, which offer more clinically relevant tissue site-specific tumour setting. Previous studies have shown that the in vivo engagement of PD-L1 blockade, CD40 ligation, and STING agonist are capable of enhancing leukaemic antigen-specific T-cell stimulatory capacity and inducing anti-leukaemic therapeutic effects in C1498 leukaemia models.^26–28^ However, these studies use highly immunogenic foreign antigen-expressing C1498 cells to validate antigen-specific anti-tumour CD8 T-cell responses and thus have potential limitations of using artificial systems. In recent years, progress on identification of neoantigens with individual specificity, which are generated by non-synonymous mutations in the tumour genome, have revealed promising preliminary clinical outcomes.^29–31^ However, though neoantigen could be a theoretically ideal target as non-self-proteins, it is frequently hard to translate a genomic mutation to a neoantigen. Moreover, single neoantigen-based personalised vaccines could only eradicate a small number of tumour cells due to the heterogeneity in tumours. Unlike neoantigens, targeting most natural tumour antigens could broaden to a wide range of cancer coverage, but they are self-proteins which are over-expressed and/or mutated in the cancerous cells while being expressed at lower levels in normal cells attributing to potential T-cell tolerance toward self-proteins. Thus, identifying targetable self-antigens (raised in tumours) and defining novel T-cell epitopes are crucial for accomplishing T-cell-mediated cancer therapy in the clinical realm, in which the same target can be used in all patients with particular malignant tumours. In this aspect, our findings provide a promising preclinical strategy in which using PLK1-derived CD8 T-cell epitope peptides one could achieve high levels of tumour-reactive T-cell responses eliciting potent therapeutic effects.

To our knowledge, the present work could be the first attempt to evaluate a peptide vaccine representing a PLK1-derived CD8 T-cell epitope. The 9-mer peptide PLK1_122_ (DSDFVFVVL) was the most effective of the predicted H-2^b^-binding candidates eliciting tumour-reactive CD8 T-cell responses in C57BL/6 mice. Interestingly, the high-scoring 8-mer PLK1_123_ (SDFVFVVL) and 10-mer PLK1_121_ (EDSDFVFVVL) in the computer-based algorithms were not as effective at inducing CD8 T-cell responses when compared to the PLK1_122_ peptide (Fig. 1a). Additionally, we included an altered peptide PLK1_345/9M_ (KGVENPLPM) to make a heteroclitic peptide vaccine, which could overcome the pre-existing immune dysfunction of cancer patients.^32^ PLK1_345/9M_ revealed slower MHC-I dissociation rates in comparison to PLK1_122_ peptide, and PLK1_345/9M_ vaccination could also evoke higher numbers of CD8 T-cells directed towards peptide-pulsed target but not effective against PLK1-expressing tumour cells (Fig. 1a, b). These results suggest that naturally processed PLK1_345_ (KGVENPLPD) may not be expressed in sufficient amounts on tumour cells to allow for T-cell recognition, whereas PLK1_122_ is a naturally processed immunodominant epitope present on the surface of various malignant tumours.

Furthermore, PLK1_122_-based vaccination could achieve higher numbers of functional and long-lasting cytotoxic CD8 T-cells capable of translating into significant therapeutic benefits against C1498 myeloid leukaemia. For these studies, we incorporated a novel vaccination regimen in which one could achieve high levels of CD8 T-cell responses using antigen-loaded DCs followed by peptide-based vaccine (TriVax) booster immunisation.^11^ Notably, PLK1_122_DC_TriVax regimen could be applied to various tumour-types to provoke tumour regression and prolonged survival, and tumour re-challenges in mice that rejected their initial tumours verified the presence of long-term antigen-specific CD8 T-cell memory immunity capable of rejecting even other types of tumour rechallenge (Fig. S3). These results suggest that PLK1 could be a shared tumour antigen that is expressed at high levels in nearly all malignant tumours and PLK1_122_-based vaccination could enable effective control over numerous tumour growths. Interestingly, PLK1_122_DC_TriVax vaccination had a superior therapeutic efficacy toward localised implanted leukaemic tumours (like a solid mass), where complete tumour rejections were observed, in comparison to its efficacy against intravenously engrafted systemic haematological leukaemia. In agreement with this, a previous report has also shown that systemic introduction of C1498 cells induced a potent T-cell tolerance caused by the abortive expansion and deletion of leukaemia-reactive CD8 T-cells, while leukaemia-specific CD8 T-cell responses were effectively induced in localised leukaemic tumour-bearing mice.^27^ These results indicate that the characteristics of tumour-reactive CD8 T-cell responses induced by PLK1_122_DC_TriVax were quite diverse depending on whether the C1498 tumour challenge was local or systemic, and more essentially, further manipulations to PLK1_122_DC_TriVax regimen are needed to circumvent T-cell tolerance and dysfunctionality that could be induced by systemic C1498 leukaemia.

It has become clear that multiple immune evasion mechanisms are present in tumour sites which potently inhibit tumour-reactive T-cell responses, leading to immune exhaustion and dysfunction eliciting tumour progression and poor clinical prognosis.^1,3,4,33^ There is evidence that combining therapeutic cancer vaccines with additional modalities such as chemotherapy and/or checkpoint inhibitors have shown synergistic therapeutic outcomes in clinic,^34^ which is not just in killing the tumour cells but in inducing a range of alterations upon cancer and/or immune cells. So far, significant advances in potentiating anti-tumour efficacy by reversing immune tolerance in tumour site have been achieved through obstruction of immune checkpoints including PD-1/PD-L1 interactions. Thus, combined therapeutic strategies with PD-1/PD-L1 checkpoint inhibitors could strengthen tumour-specific immune responses though they may not work in all individuals and in all cancers. We and other have shown that PD-1/PD-L1 blockade prevents exhaustion of tumour-reactive T-cells even in haematological malignancies, leading to augmented effector function and persistence of antigen-specific T-cells at the tumour site.^10,35,36^ Indeed, we observed that the systemic administration of anti-PD-L1 Abs with PLK1_122_DC_TriVax led to an increase in the frequency of tumour-reactive CD8 T-cells that had higher antigen-recognition functionality, resulting in enhanced anti-tumour efficacy with an increase of overall T-cell numbers in systemic C1498 leukaemia (Fig. 3). Importantly, prolonged intervention of anti-PD-L1, not anti-PD-1, resulted in increase of therapeutic effectiveness of PLK1_122_DC_TriVax where complete tumour rejections were observed (Fig. S5). Although our results are in line with previous reports that demonstrate the synergistic effects of immune checkpoint inhibitors with vaccines,^10,35,37^ we believe that there is a clear need for additional strategies to optimise PLK1_122_DC_TriVax vaccination regimen to achieve the desired therapeutic benefits.

It is also evident that tumour cells avoid immune recognition through selective outgrowth of new subclones that are defective on the expression of immunogenic antigens and/or the antigen-presenting machinery, leading to become more heterogeneous subsets in a wide range of tumours.^38–40^ Particularly, previous studies demonstrated that myeloid disorders including acute leukaemia and myelodysplastic syndromes exhibit clonal heterogeneity that evolves upon disease progression and/or relapse.^20–22^ In our studies, remarkable therapeutic efficacy (marked by several complete tumour eradications) was accomplished with PLK1_122_DC_TriVax vaccination under PD-L1 blockade in clonally homogenous C1498^Homo^-engrafted leukaemic mice (Fig. 4). In contrast, although intensely reduced tumour progressions and improved survival rates were accomplished in clonally heterogeneous C1498-luc-engrafted leukaemic mice, no complete tumour regressions were observed (Fig. 3). These results support that tumour sub-clonal heterogeneity in target antigen expression is a major obstacle in developing therapeutic cancer vaccines. Recently, targeting CD19-expressing tumours with anti-CD19-chimeric antigen receptor (CAR)-T-cells have obtained considerable clinical outcomes toward B-cell malignancies, including complete remissions.^41,42^ However, low and/or loss of CD19 expression and outgrowth of CD19-negative tumour variants have been reported in both paediatric and adult responders following CD19-CAR-T-cell therapy.^41,43,44^ To compensate these circumstances, dual-antigen-targeting CAR-T-cells with two CAR constructs have developed to overcome the outgrowth of antigen-loss variants because the chance of simultaneous loss of two antigens is relatively low.^45,46^ Similarly, our results showed that multi-peptide-loaded DCs prime_TriVax boost strategies allowed the simultaneous induction of CD8 T-cell responses to multiple epitopes (Fig. 5). Moreover, DC_TriVax vaccination targeting PLK1 and Sur simultaneously led to evidently enhanced therapeutic benefits in comparison to DC_TriVax using each individual peptide even in clonally heterogeneous C1498-engrafted leukaemic mice. More importantly, the combinations of sustained PD-L1 blockade to the PLK1_122_/Sur_20_DC_TriVax resulted in remarkable enhanced anti-tumour effects, in which the majority of the mice achieved complete tumour eradication of heterogeneous C1498 myeloid leukaemia (Fig. 6). Our results conclude that peptide vaccination regimen capable of inducing concurrent multivalent CD8 T-cell responses specific to more than one antigen could circumvent the potential drawbacks of single-peptide-based vaccines, and that the sufficient endurance of PD1 blockade amplified significantly the effectiveness of cancer vaccines comprising multi-peptides as well as single-peptide.

The clinical outcomes of cancer vaccine therapies notwithstanding, potential concerns have been raised over targeting the overexpressed tumour antigens because they also show a low level of expression in some healthy tissues, which may induce unknown and possibly hazardous self-reactive adverse effect.^47^ In view of this, neoantigens with stronger immunogenicity are a theoretically ideal target as non-self-proteins, which would not evoke potential autoimmunity in cancer patients.^30,31^ However, large differences among tumour types and individuals of neoantigens, and long preparation periods on neoantigen-based therapeutics extremely limit the clinical application. Although unexpected adverse events may occur when antigen-specific T-cells fail to discriminate levels of tumour antigens presented on normal versus tumour cells, and/or memory T-cell responses are reactivated by vaccinations, autologous vaccination with peptides derived from survivin and telomerase reverse transcriptase (TERT), which are overexpressed in the vast majority of human cancers with minimal expression in normal tissue, has proven effective in inducing antigen-specific T cell precursors and safe indicating no severe adverse events.^48^ Moreover, even in studies using TCR-engineered redirected T cells with high-avidity TCR specific for survivin and TERT, severe auto-reactive toxicity was not reported.^49,50^ As PLK1 protein is essential for cell-cycle regulation, they could be detected in highly proliferating normal tissues, targeting PLK1 antigen may occur potential adverse events. Nonetheless, we observed that the freshly isolated CD8 T cells from PLK1_122_DC_TriVax-vaccinated mice do not recognise normal liver cells, and the mice did not show common autoimmune pathophysiological symptoms such as weight change and abnormal behaviours. These observations imply that targeting PLK1 protein in immunotherapeutic strategies has a potential anti-tumour application in the future though it needs to be further investigated.

Collectively, we present data demonstrating that combined immunotherapeutic strategy of multi-antigenic cancer vaccines with sustained PD-L1 blockade are able to induce potent tumour-reactive T-cells capable of overcoming the potential immune escaping of clonally heterogeneous myeloid leukaemia, resulting in remarkable therapeutic anti-tumour benefits. Additional studies with a focus on optimising the peptide vaccination regime that affect the specificity and avidity of tumour-reactive T-cells towards tumour escaping variants causing clonal heterogeneity could facilitate clinical applications in the treatment of various tumours, including haematologic malignancies.

## Supplementary Information

Supplementary data

## Data Availability

Summarised primary research data are presented in the paper. No publicly available dataset has been generated as part of this work.
