# Supplementary material for: An effective peptide vaccine strategy circumventing clonal MHC heterogeneity of murine myeloid leukaemia
Source: Br J Cancer. 2020 Jun 29;123(6):919–31. doi: 10.1038/s41416-020-0955-y (PMC7492404; doi:10.1038/s41416-020-0955-y)
Supplement: Supplementary file 1 — Supplementary data [file 41416_2020_955_MOESM1_ESM.pptx]

## Slide 1
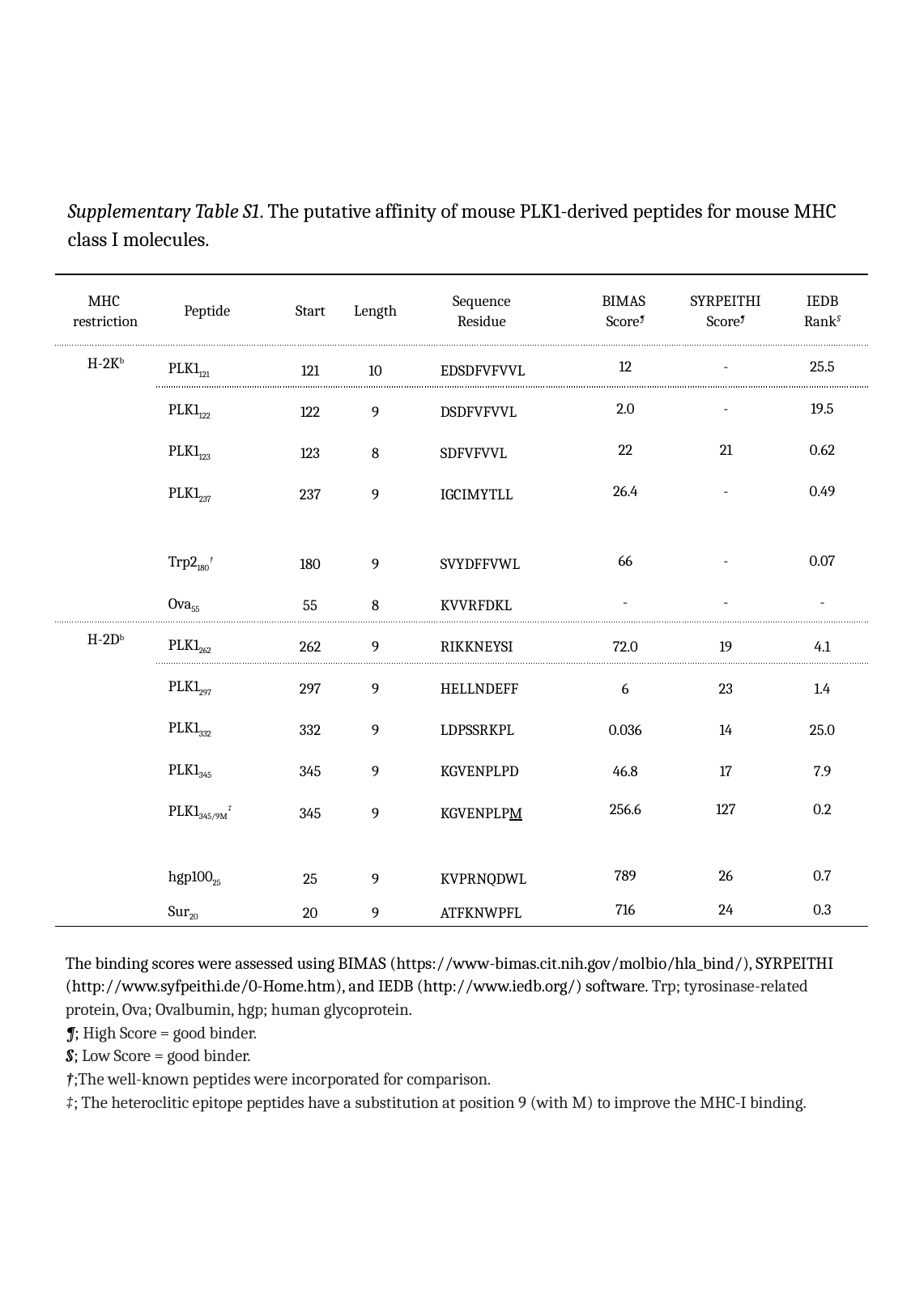

Supplementary Table S1. The putative affinity of mouse PLK1-derived peptides for mouse MHC class I molecules.
| MHC restriction | Peptide | | Start | Length | Sequence Residue | | BIMAS Score¶ | SYRPEITHI Score¶ | IEDB Rank§ |
| --- | --- | --- | --- | --- | --- | --- | --- | --- | --- |
| H-2Kb | PLK1121 | | 121 | 10 | EDSDFVFVVL | | 12 | - | 25.5 |
| | PLK1122 | | 122 | 9 | DSDFVFVVL | | 2.0 | - | 19.5 |
| | PLK1123 | | 123 | 8 | SDFVFVVL | | 22 | 21 | 0.62 |
| | PLK1237 | | 237 | 9 | IGCIMYTLL | | 26.4 | - | 0.49 |
| | | | | | | | | | |
| | Trp2180† | | 180 | 9 | SVYDFFVWL | | 66 | - | 0.07 |
| | Ova55 | | 55 | 8 | KVVRFDKL | | - | - | - |
| H-2Db | PLK1262 | | 262 | 9 | RIKKNEYSI | | 72.0 | 19 | 4.1 |
| | PLK1297 | | 297 | 9 | HELLNDEFF | | 6 | 23 | 1.4 |
| | PLK1332 | | 332 | 9 | LDPSSRKPL | | 0.036 | 14 | 25.0 |
| | PLK1345 | | 345 | 9 | KGVENPLPD | | 46.8 | 17 | 7.9 |
| | PLK1345/9M‡ | | 345 | 9 | KGVENPLPM | | 256.6 | 127 | 0.2 |
| | | | | | | | | | |
| | hgp10025 | | 25 | 9 | KVPRNQDWL | | 789 | 26 | 0.7 |
| | Sur20 | | 20 | 9 | ATFKNWPFL | | 716 | 24 | 0.3 |
The binding scores were assessed using BIMAS (https://www-bimas.cit.nih.gov/molbio/hla_bind/), SYRPEITHI (http://www.syfpeithi.de/0-Home.htm), and IEDB (http://www.iedb.org/) software. Trp; tyrosinase-related protein, Ova; Ovalbumin, hgp; human glycoprotein.
¶; High Score = good binder.
§; Low Score = good binder.
†;The well-known peptides were incorporated for comparison.
‡; The heteroclitic epitope peptides have a substitution at position 9 (with M) to improve the MHC-I binding.

## Slide 2
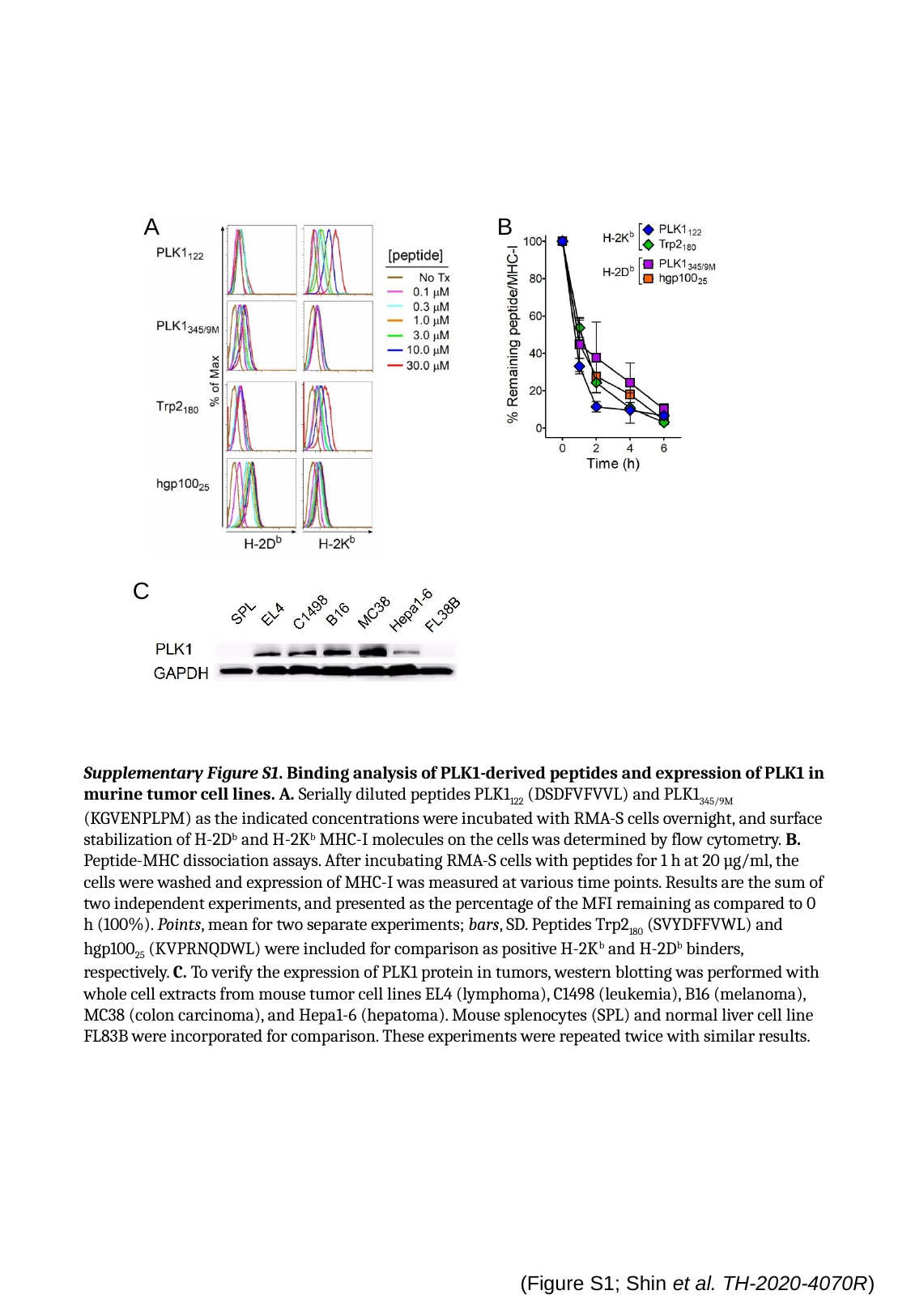

A
B
C
Supplementary Figure S1. Binding analysis of PLK1-derived peptides and expression of PLK1 in murine tumor cell lines. A. Serially diluted peptides PLK1122 (DSDFVFVVL) and PLK1345/9M (KGVENPLPM) as the indicated concentrations were incubated with RMA-S cells overnight, and surface stabilization of H-2Db and H-2Kb MHC-I molecules on the cells was determined by flow cytometry. B. Peptide-MHC dissociation assays. After incubating RMA-S cells with peptides for 1 h at 20 µg/ml, the cells were washed and expression of MHC-I was measured at various time points. Results are the sum of two independent experiments, and presented as the percentage of the MFI remaining as compared to 0 h (100%). Points, mean for two separate experiments; bars, SD. Peptides Trp2180 (SVYDFFVWL) and hgp10025 (KVPRNQDWL) were included for comparison as positive H-2Kb and H-2Db binders, respectively. C. To verify the expression of PLK1 protein in tumors, western blotting was performed with whole cell extracts from mouse tumor cell lines EL4 (lymphoma), C1498 (leukemia), B16 (melanoma), MC38 (colon carcinoma), and Hepa1-6 (hepatoma). Mouse splenocytes (SPL) and normal liver cell line FL83B were incorporated for comparison. These experiments were repeated twice with similar results.
(Figure S1; Shin et al. TH-2020-4070R)

## Slide 3
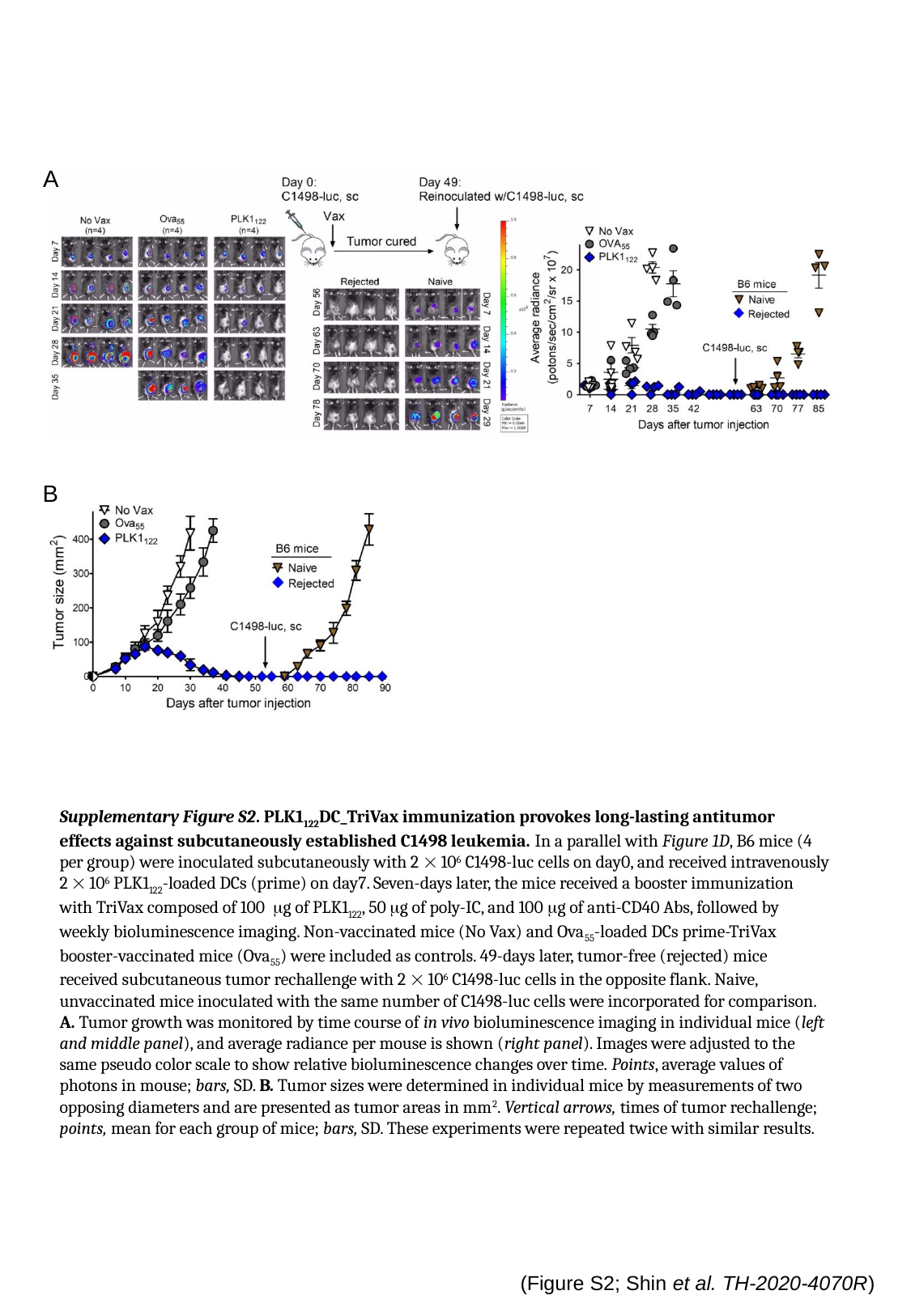

A
B
Supplementary Figure S2. PLK1122DC_TriVax immunization provokes long-lasting antitumor effects against subcutaneously established C1498 leukemia. In a parallel with Figure 1D, B6 mice (4 per group) were inoculated subcutaneously with 2 × 10^6^ C1498-luc cells on day0, and received intravenously 2 × 10^6^ PLK1122-loaded DCs (prime) on day7. Seven-days later, the mice received a booster immunization with TriVax composed of 100 μg of PLK1122, 50 μg of poly-IC, and 100 μg of anti-CD40 Abs, followed by weekly bioluminescence imaging. Non-vaccinated mice (No Vax) and Ova55-loaded DCs prime-TriVax booster-vaccinated mice (Ova55) were included as controls. 49-days later, tumor-free (rejected) mice received subcutaneous tumor rechallenge with 2 × 10^6^ C1498-luc cells in the opposite flank. Naive, unvaccinated mice inoculated with the same number of C1498-luc cells were incorporated for comparison. A. Tumor growth was monitored by time course of in vivo bioluminescence imaging in individual mice (left and middle panel), and average radiance per mouse is shown (right panel). Images were adjusted to the same pseudo color scale to show relative bioluminescence changes over time. Points, average values of photons in mouse; bars, SD. B. Tumor sizes were determined in individual mice by measurements of two opposing diameters and are presented as tumor areas in mm2. Vertical arrows, times of tumor rechallenge; points, mean for each group of mice; bars, SD. These experiments were repeated twice with similar results.
(Figure S2; Shin et al. TH-2020-4070R)

## Slide 4
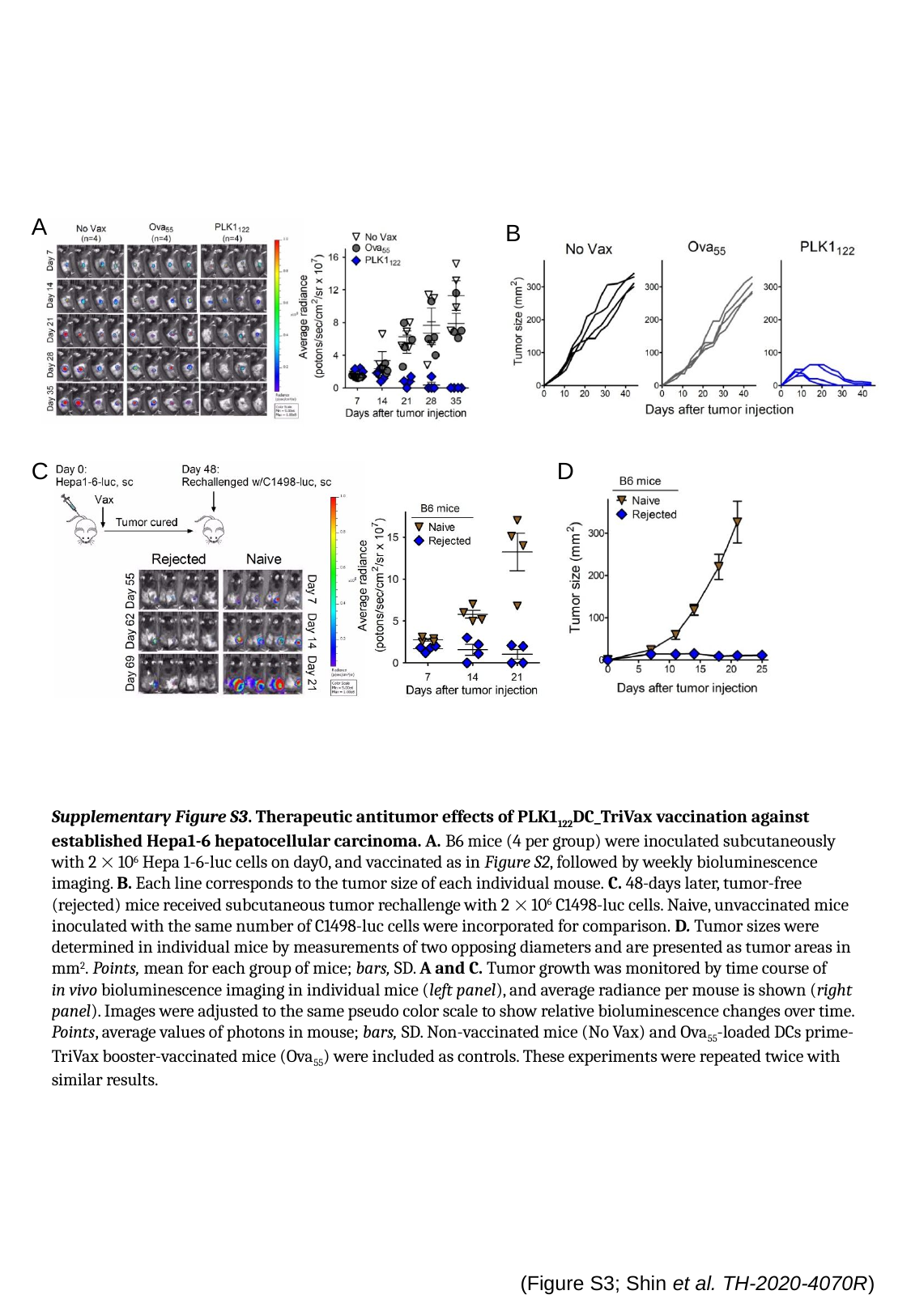

A
B
C
D
Supplementary Figure S3. Therapeutic antitumor effects of PLK1122DC_TriVax vaccination against established Hepa1-6 hepatocellular carcinoma. A. B6 mice (4 per group) were inoculated subcutaneously with 2 × 10^6^ Hepa 1-6-luc cells on day0, and vaccinated as in Figure S2, followed by weekly bioluminescence imaging. B. Each line corresponds to the tumor size of each individual mouse. C. 48-days later, tumor-free (rejected) mice received subcutaneous tumor rechallenge with 2 × 10^6^ C1498-luc cells. Naive, unvaccinated mice inoculated with the same number of C1498-luc cells were incorporated for comparison. D. Tumor sizes were determined in individual mice by measurements of two opposing diameters and are presented as tumor areas in mm2. Points, mean for each group of mice; bars, SD. A and C. Tumor growth was monitored by time course of in vivo bioluminescence imaging in individual mice (left panel), and average radiance per mouse is shown (right panel). Images were adjusted to the same pseudo color scale to show relative bioluminescence changes over time. Points, average values of photons in mouse; bars, SD. Non-vaccinated mice (No Vax) and Ova55-loaded DCs prime-TriVax booster-vaccinated mice (Ova55) were included as controls. These experiments were repeated twice with similar results.
(Figure S3; Shin et al. TH-2020-4070R)

## Slide 5
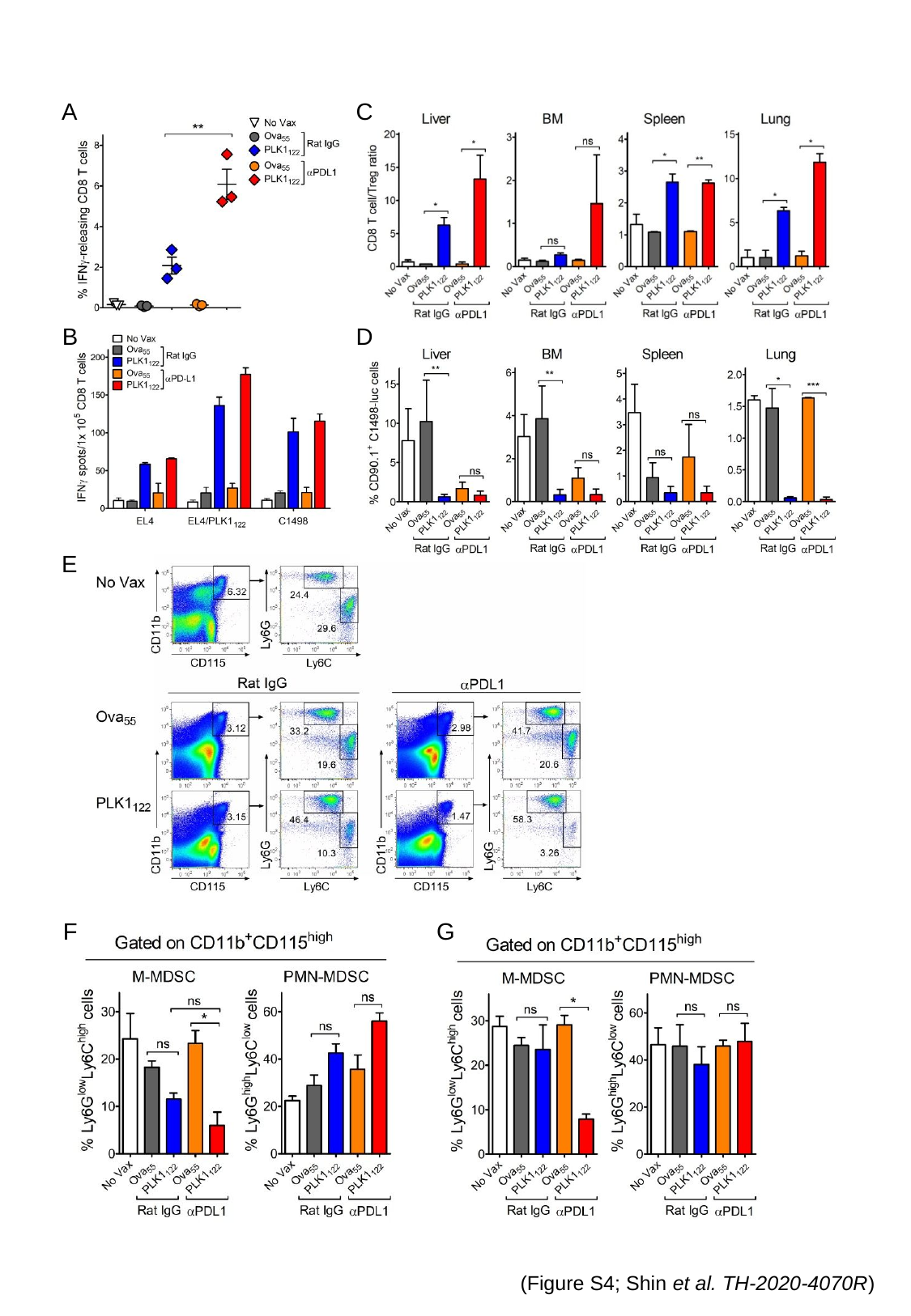

A
C
B
D
E
F
G
(Figure S4; Shin et al. TH-2020-4070R)

## Slide 6
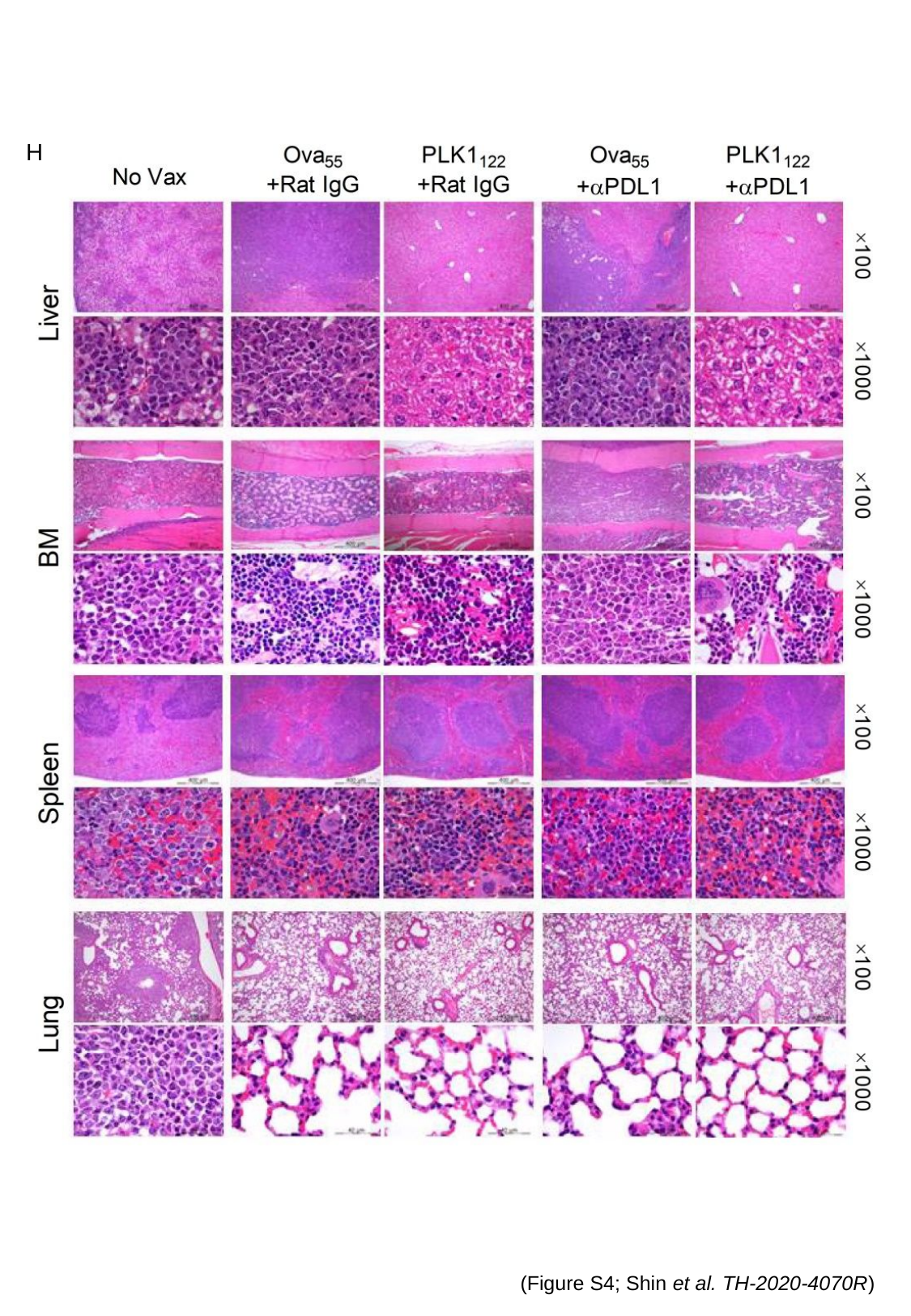

H
(Figure S4; Shin et al. TH-2020-4070R)

## Slide 7
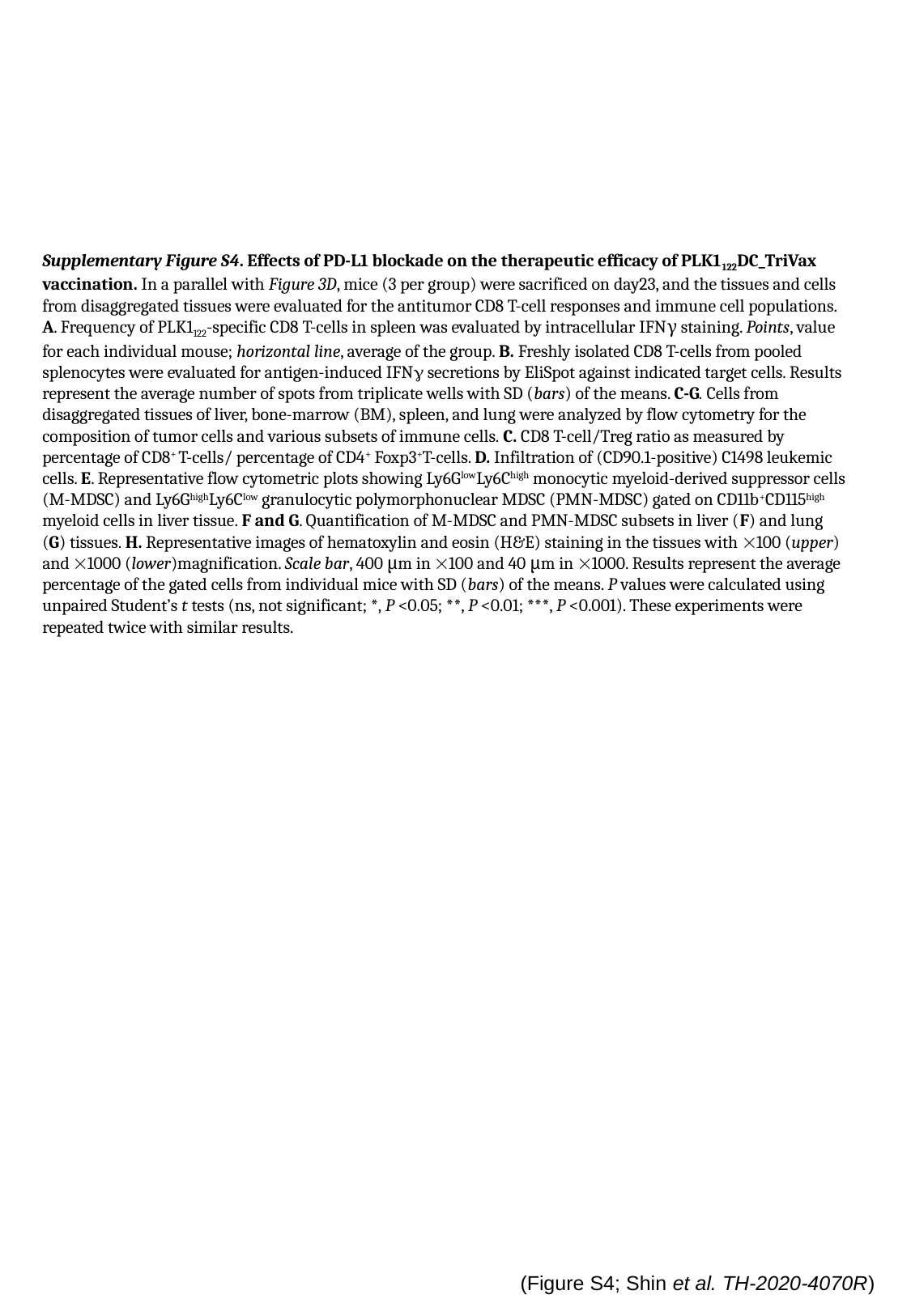

Supplementary Figure S4. Effects of PD-L1 blockade on the therapeutic efficacy of PLK1122DC_TriVax vaccination. In a parallel with Figure 3D, mice (3 per group) were sacrificed on day23, and the tissues and cells from disaggregated tissues were evaluated for the antitumor CD8 T-cell responses and immune cell populations. A. Frequency of PLK1122-specific CD8 T-cells in spleen was evaluated by intracellular IFNγ staining. Points, value for each individual mouse; horizontal line, average of the group. B. Freshly isolated CD8 T-cells from pooled splenocytes were evaluated for antigen-induced IFNγ secretions by EliSpot against indicated target cells. Results represent the average number of spots from triplicate wells with SD (bars) of the means. C-G. Cells from disaggregated tissues of liver, bone-marrow (BM), spleen, and lung were analyzed by flow cytometry for the composition of tumor cells and various subsets of immune cells. C. CD8 T-cell/Treg ratio as measured by percentage of CD8+ T-cells/ percentage of CD4+ Foxp3+T-cells. D. Infiltration of (CD90.1-positive) C1498 leukemic cells. E. Representative flow cytometric plots showing Ly6GlowLy6Chigh monocytic myeloid-derived suppressor cells (M-MDSC) and Ly6GhighLy6Clow granulocytic polymorphonuclear MDSC (PMN-MDSC) gated on CD11b+CD115high myeloid cells in liver tissue. F and G. Quantification of M-MDSC and PMN-MDSC subsets in liver (F) and lung (G) tissues. H. Representative images of hematoxylin and eosin (H&E) staining in the tissues with ×100 (upper) and ×1000 (lower)magnification. Scale bar, 400 μm in ×100 and 40 μm in ×1000. Results represent the average percentage of the gated cells from individual mice with SD (bars) of the means. P values were calculated using unpaired Student’s t tests (ns, not significant; *, P <0.05; **, P <0.01; ***, P <0.001). These experiments were repeated twice with similar results.
(Figure S4; Shin et al. TH-2020-4070R)

## Slide 8
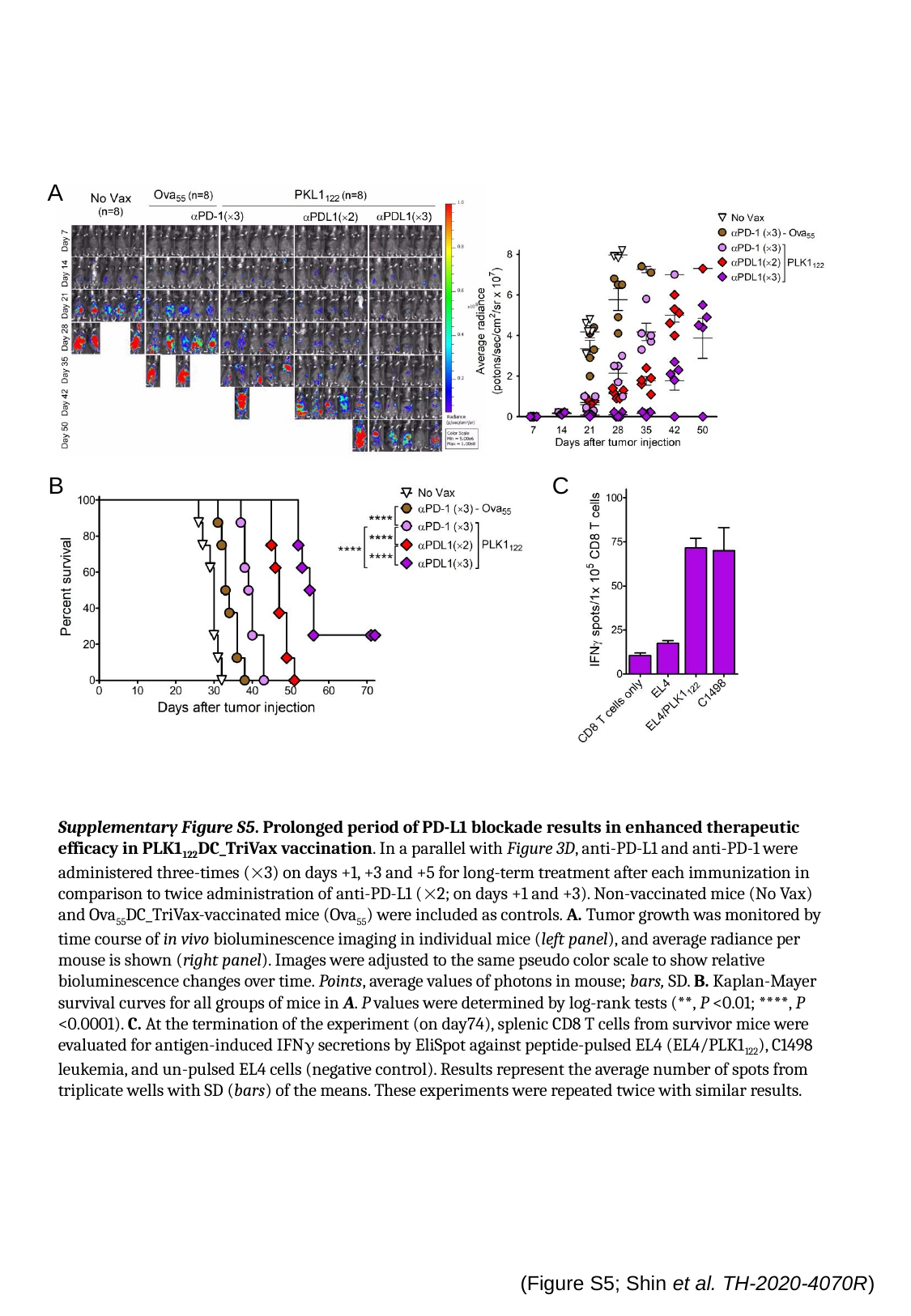

A
B
C
Supplementary Figure S5. Prolonged period of PD-L1 blockade results in enhanced therapeutic efficacy in PLK1122DC_TriVax vaccination. In a parallel with Figure 3D, anti-PD-L1 and anti-PD-1 were administered three-times (×3) on days +1, +3 and +5 for long-term treatment after each immunization in comparison to twice administration of anti-PD-L1 (×2; on days +1 and +3). Non-vaccinated mice (No Vax) and Ova55DC_TriVax-vaccinated mice (Ova55) were included as controls. A. Tumor growth was monitored by time course of in vivo bioluminescence imaging in individual mice (left panel), and average radiance per mouse is shown (right panel). Images were adjusted to the same pseudo color scale to show relative bioluminescence changes over time. Points, average values of photons in mouse; bars, SD. B. Kaplan-Mayer survival curves for all groups of mice in A. P values were determined by log-rank tests (**, P <0.01; ****, P <0.0001). C. At the termination of the experiment (on day74), splenic CD8 T cells from survivor mice were evaluated for antigen-induced IFNγ secretions by EliSpot against peptide-pulsed EL4 (EL4/PLK1122), C1498 leukemia, and un-pulsed EL4 cells (negative control). Results represent the average number of spots from triplicate wells with SD (bars) of the means. These experiments were repeated twice with similar results.
(Figure S5; Shin et al. TH-2020-4070R)

## Slide 9
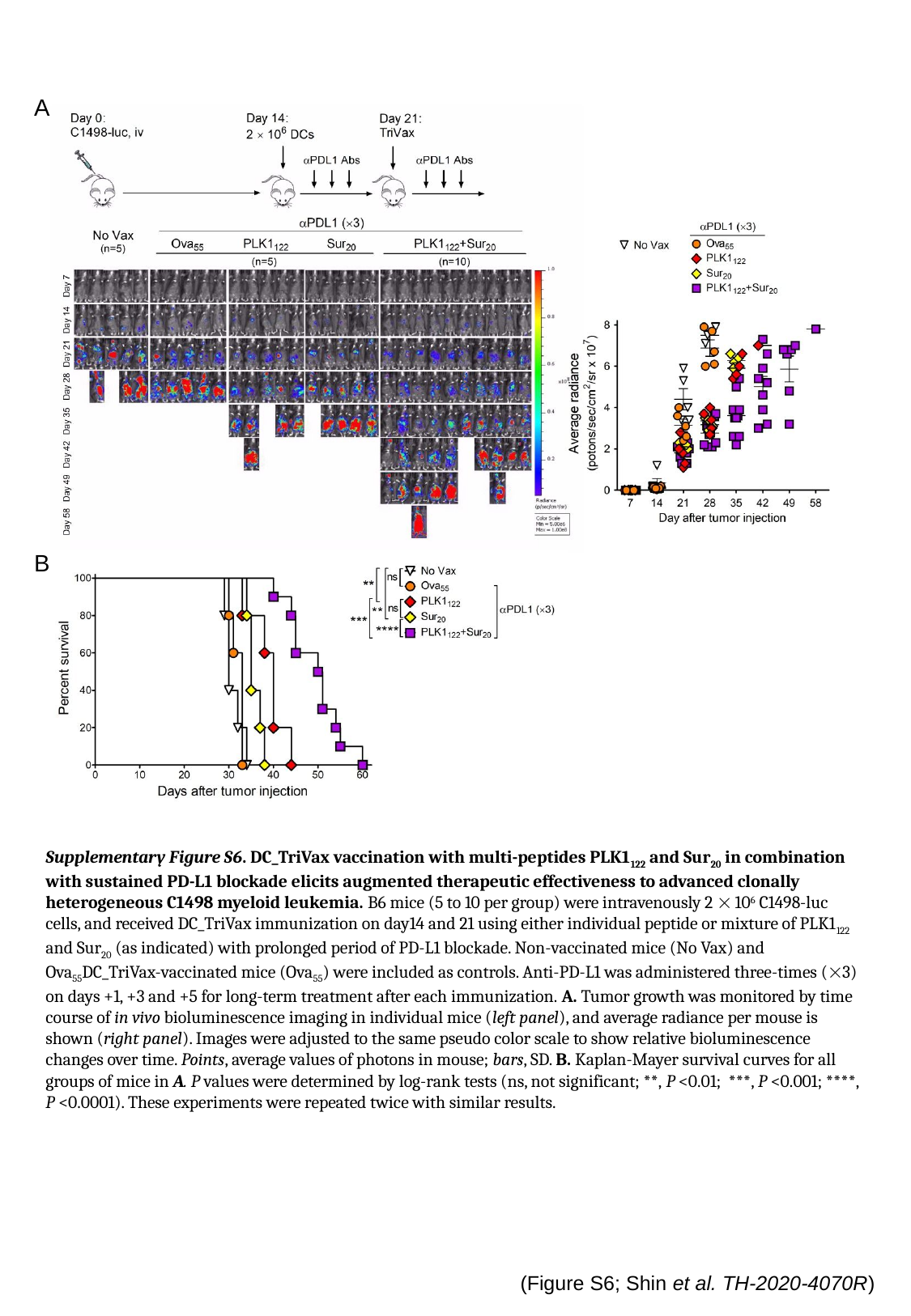

A
B
Supplementary Figure S6. DC_TriVax vaccination with multi-peptides PLK1122 and Sur20 in combination with sustained PD-L1 blockade elicits augmented therapeutic effectiveness to advanced clonally heterogeneous C1498 myeloid leukemia. B6 mice (5 to 10 per group) were intravenously 2 × 10^6^ C1498-luc cells, and received DC_TriVax immunization on day14 and 21 using either individual peptide or mixture of PLK1122 and Sur20 (as indicated) with prolonged period of PD-L1 blockade. Non-vaccinated mice (No Vax) and Ova55DC_TriVax-vaccinated mice (Ova55) were included as controls. Anti-PD-L1 was administered three-times (×3) on days +1, +3 and +5 for long-term treatment after each immunization. A. Tumor growth was monitored by time course of in vivo bioluminescence imaging in individual mice (left panel), and average radiance per mouse is shown (right panel). Images were adjusted to the same pseudo color scale to show relative bioluminescence changes over time. Points, average values of photons in mouse; bars, SD. B. Kaplan-Mayer survival curves for all groups of mice in A. P values were determined by log-rank tests (ns, not significant; **, P <0.01; ***, P <0.001; ****, P <0.0001). These experiments were repeated twice with similar results.
(Figure S6; Shin et al. TH-2020-4070R)
